# Antibiofilm Activity of *Agrimonia eupatoria* Extracts Against Clinically Relevant Pathogens

**DOI:** 10.1155/ijm/5222416

**Published:** 2026-02-20

**Authors:** Jelena N. Terzić, Aleksandar G. Kočović, Marina M. Stanković, Olgica D. Stefanović

**Affiliations:** ^1^ Department of Biology and Ecology, Faculty of Science, University of Kragujevac, Kragujevac, Serbia, kg.ac.rs; ^2^ Department of Pharmacy, Faculty of Medical Sciences, University of Kragujevac, Kragujevac, Serbia, kg.ac.rs; ^3^ Center of Excellence for Redox Balance Research in Cardiovascular and Metabolic Disorders, Faculty of Medical Sciences, University of Kragujevac, Kragujevac, Serbia, kg.ac.rs

**Keywords:** *Agrimonia eupatoria*, antibiofilm activity, bacterial motility, biofilm, phytochemical analysis, wound healing

## Abstract

Biofilms are surface‐attached bacterial communities that contribute significantly to chronic infections. Their altered metabolism promotes antibiotic resistance and makes treatment more difficult. Alternative strategies, such as the use of medicinal plants, are being actively investigated and *Agrimonia eupatoria* L. is one of them. This study is aimed at evaluating the antibiofilm activity of ethanol, acetone, and ethyl acetate extracts of *A. eupatoria* against *Staphylococcus aureus*, *Proteus* spp., and *Pseudomonas aeruginosa* strains isolated from human wounds. In addition, the effects of these extracts on bacterial auto‐aggregation, surface hydrophobicity, and motility were investigated. The phytochemical analysis included FT‐IR spectroscopy and spectrophotometric quantification of the total phenolic compounds. FT‐IR analysis confirmed the presence of characteristic functional groups vibrations (O–H at ~3425 cm^−1^, C=O at ~1735–1685 cm^−1^, and aromatic C=C at ~1618–1515 cm^−1^), whereas phytochemical profiling revealed that the ethyl acetate extract contained the highest phenolic acid content (149.65 ± 0.73 mg CAE/g). All tested extracts inhibited initial cell adhesion and biofilm formation, although they were less effective against mature biofilms. Among them, acetone and ethyl acetate extracts exhibited the strongest antibiofilm activity. *S. aureus* strains S1 and S2 were the most susceptible, with inhibition rates of at least 92% and 85% for the acetone extract, and 78% and 86% for the ethyl acetate extract, respectively. Although auto‐aggregation and cell surface hydrophobicity remained unaffected, both swimming and swarming motilities were reduced. Finally, fluorescence microscopy confirmed that the inhibitory effect was dose‐dependent. These results suggest that *A. eupatoria* extracts represent promising natural antibiofilm agents against clinically relevant human pathogens.

## 1. Introduction

Bacterial biofilms are structured assemblages of cells that adhere to surfaces or/and to each other and represent a bacterial survival strategy [1, 2]. In biofilms, bacteria are embedded in a self‐producing matrix of extracellular polymeric substances (EPSs) consisting mainly of extracellular DNA, proteins, and polysaccharides. Biofilm‐forming bacteria have the ability to colonize and grow on biotic or abiotic surfaces such as living tissue and implanted medical devices [3]. In biofilms, bacteria exhibit phenotypes that differ from those of free‐living planktonic cells [4]. They show a much higher tolerance to the host′s immune defense and antimicrobial agents [3]. Bacterial biofilms contribute to persistent, hard‐to‐treat infections. It is estimated that biofilms are responsible for almost 80% of all human bacterial infections, underscoring their significance as a virulence factor [5].

This problem, along with bacterial resistance to antibiotics, emphasizes the need to find new, alternative treatments. The use of naturally occurring compounds from medicinal plants is one potential approach [6–8]. There is a long history of using plants in nutrition and as medicines to treat various diseases. Plants are a great source of secondary metabolites such as phenolic compounds (e.g., flavonoids and tannins), terpenes and steroids (e.g., monoterpenes, sesquiterpenes, and diterpenes), and nitrogen‐containing compounds (e.g., alkaloids), which are widely used in the pharmaceutical industry [9]. It is already known that some plant compounds can prevent the attachment of bacterial cells and the formation of biofilms [8, 10–12].

*Agrimonia eupatoria* L. (also known as agrimony) is a perennial herbaceous plant from the Rosaceae family [13]. Due to its favorable properties, it is often used in traditional medicine in Europe to treat bronchitis and sore throats, urinary tract infections, gastrointestinal diseases, and chronic wounds [14]. The biological effects of *A. eupatoria* are associated with the presence of tannins, flavonoids, phenolic acids, and terpenoids [13].

The antimicrobial activity of *A. eupatoria* has been documented in studies that emphasized its effectiveness against various bacterial species. In the study by Muruzović et al. [15], the antibacterial activity of water, diethyl ether, acetone, and ethanol extracts of *A. eupatoria* was determined. The results obtained depended on the type of extract (diethyl ether < ethanol < water < acetone) and the species of bacteria; the effect was weaker on the gram‐negative bacteria than on gram‐positive bacteria. Ghaima [16] showed that the ethanol extract of *A. eupatoria* was more effective than the aqueous extract using the agar diffusion method. The highest inhibition was observed with ethanol extract against *Escherichia coli* at a concentration of 10 mg/mL. On the other hand, the ethanol extract showed moderate antibacterial activity against *Pseudomonas aeruginosa* and *Staphylococcus aureus* at the same concentration. In addition to its antibacterial properties, *A. eupatoria* is often used in wound healing due to its bioactive compounds, such as tannins and flavonoids, which have antioxidant and immunomodulatory properties. Topical application of *A. eupatoria* extracts has been shown to accelerate wound healing by promoting collagen formation and granulation tissue development while reducing bacterial colonization, particularly by *S. aureus*, a common pathogen of wound infections. In addition, the antimicrobial effect of the plant may help in the treatment of chronic skin conditions such as psoriasis and eczema, where microbial colonization exacerbates symptoms [14]. These results emphasize the potential of *A. eupatoria* as a natural remedy for wound care and infection management.

The antibiofilm activity of *A. eupatoria* has not yet been studied in detail. Muruzović et al. [15] demonstrated the ability of water and acetone extracts of *A. eupatoria* to prevent biofilm formation of *P. aeruginosa* and *Proteus mirabilis*. However, no studies were conducted on the different phases of biofilm formation, bacterial motility, auto‐aggregation, or cell hydrophobicity as factors associated with biofilm development.

In view of all this, this study is aimed at investigating the antibiofilm activity of ethanol, acetone, and ethyl acetate extracts of *A. eupatoria* on bacterial strains isolated from human wounds. We also investigated the effects of the extracts on bacterial motility, auto‐aggregation, and cell hydrophobicity. In addition, a phytochemical analysis of the plant extracts was performed.

## 2. Materials and Methods

### 2.1. Plant Material and Extraction

Dried aerial parts of *A. eupatoria* were provided by the Institute of Medicinal Plants “Dr Josif Pančić”, Belgrade, Serbia. The plant material was extracted with ethanol, acetone, or ethyl acetate using ultrasound‐assisted extraction as previously described [17]. A total of 26 g of the plant material per extract was soaked with 800 mL of solvent. After extraction and filtration, the solvent was evaporated using a rotary evaporator (RV10 basic, IKA, Bensheim, Germany) at low pressure and low temperature (40°C) to obtain dry extracts. The dry extracts were stored at −20°C until use.

### 2.2. Phytochemical Analysis

#### 2.2.1. Fourier Transform Infrared (FT‐IR) Spectroscopy

FT‐IR spectroscopy was used to identify chemical bond types (functional groups) of extracted compounds based on the peak values in the infrared radiation region. Small amounts of dry powder of the extracts were separately encapsulated in a KBr pellet to prepare translucent sample disks. The samples were analyzed using an FT‐IR spectrometer (PerkinElmer, IR, Waltham, United States) with a scanning range from 450 to 4000 cm^−1^ with a resolution of 4 cm^−1^. Reference FT‐IR spectra of representative phenolic standards (hesperidin, rutin, quercetin·H₂O, gallic acid, syringic acid, caffeic acid, *trans*‐ferulic acid, and protocatechuic acid) were recorded under identical instrumental conditions to support assignment of vibrational regions.

#### 2.2.2. Determination of Phenolic Compounds Content

To determine the content of phenolic compounds, the extracts were dissolved in methanol (> 99.8%, Sigma‐Aldrich Co., St. Louis, United States) to achieve a concentration of 1 mg/mL.

Thus, the obtained extract solution was analyzed to determine the total phenolic content (TPC) using the Folin–Ciocalteu method described by Wootton‐Beard et al. [18]. The results were expressed as gallic acid equivalents (mg GAE/g of extract). The total phenolic acid (TPA) content was determined according to the method described by Gawlik‐Dziki [19], and the results were expressed as caffeic acid equivalents (mg CAE/g of extract). The aluminum chloride method described by Quettier‐Deleu et al. [20] was used to quantify the total flavonoid content (TFC), and the results were expressed as rutin equivalents (mg RUE/g of extract). The total proanthocyanidin content (TPAC) was determined as described by Hagerman et al. [21] and the results were expressed as equivalents of cyanidin chloride (mg CChE/g extract). All determinations were carried out in triplicate.

#### 2.2.3. Chemometric Analysis

Chemometric analysis of the FT‐IR spectra was performed using principal component analysis (PCA) and hierarchical cluster analysis (HCA) in IBM SPSS Statistics (Version 21.0, IBM Corp., Armonk, New York, United States). FT‐IR spectra of *A. eupatoria* extracts and phenolic standards were first converted from transmittance (*%* *T*) to absorbance according to Equation (1):

(1) A=log10100%T.

All spectra were interpolated onto a shared wavenumber grid defined by the intersection of all datasets to ensure direct comparability. Two data matrices were constructed: extracts only and extracts combined with authentic phenolic standards (gallic, protocatechuic, caffeic, syringic, and *trans*‐ferulic acids and hesperidin, rutin, and quercetin hydrate). Chemometric analyses were performed on both the full FT‐IR spectral range (4000–450 cm^−1^) and the fingerprint region (1800–650 cm^−1^), which is particularly informative for phenolic compound discrimination [22]. Prior to multivariate analysis, all variables were mean‐centered and autoscaled (unit variance scaling) to remove intensity‐driven bias and to equalize the contribution of all spectral regions [23]. PCA was conducted using the covariance matrix with eigenvalue extraction and varimax rotation, and factor scores were saved for subsequent visualization. The first principal components were extracted based on eigenvalues greater than unity, and factor scores were saved for subsequent visualization. HCA was performed on the standardized absorbance matrix using Ward′s linkage method and Euclidean distance [24]. Dendrograms were generated to visualize the clustering patterns and assess similarities among samples based on their spectral profiles. The observed shifts in sample positions between PCA models with and without standards reflect the intrinsic orthogonal axis reorientation determined solely by the variance structure of the dataset [25, 26].

#### 2.2.4. Bacterial Strains

In this study, nine bacterial strains isolated from human wounds and two reference strains from the American Type Culture Collection (ATCC) were selected. Four strains of gram‐positive bacterium *S. aureus* (S1, S2, S3, and S4) and five strains of gram‐negative bacteria *Proteus* spp. (Pr1) and *P. aeruginosa* (PA1, PA2, PA3, and PA4) were generously provided by the Microbiology Laboratory of the Paraćin Hospital, Serbia. Identification was performed using blood agar (Torlak, Belgrade, Serbia), HiChrome UTI Agar (HiMedia Laboratories, Mumbai, India), biochemical tests, and gram staining. Reference strains *S. aureus* ATCC 25923 and *P. aeruginosa* ATCC 10145 were purchased from Sigma‐Aldrich, St. Louis, United States. The bacterial strains were kept in 20% glycerol/medium stock at −80°C. Prior to testing, the bacterial strains were subcultured twice on nutrient agar (Torlak, Belgrade, Serbia) at 37°C for 18 h. All tested strains were strong biofilm producers, which was confirmed in our previous study [27].

### 2.3. Determination of Minimum Inhibitory Concentrations (MICs)

The MIC of *A. eupatoria* extracts was determined using the broth microdilution method in Mueller–Hinton broth (HiMedia Laboratories, Mumbai, India) [28]. The extracts were serially diluted to obtain a concentration range of 0.156–10 mg/mL. The final bacterial inoculum utilized in each well was approximately 5 × 10^5^ colony‐forming units (CFU)/mL. Bacterial growth was observed by adding resazurin (Alfa Aesar GmbH & Co., Karlsruhe, Germany), an indicator of microbial growth. When viable cells reduce resazurin to resorufin, the blue, nonfluorescent dye changes to pink, fluorescent dye. The lowest concentration of the tested plant extract that prevented resazurin color change from blue to pink was determined as the MIC [29]. The stock solutions of plant extracts were prepared in 10% DMSO (Sigma‐Aldrich Co., St. Louis, United States). Each experiment included a growth control (broth + bacterium), a sterility control (broth + extract), and a solvent control (10% DMSO). Tetracycline (Sigma‐Aldrich Co., St. Louis, United States) dissolved in nutrient broth was used as a positive control. The concentration range examined was from 0.0005 to 0.128 mg/mL.

### 2.4. Determination of Antibiofilm Activity

#### 2.4.1. Biofilm Formation Inhibition: Cell Attachment and Biofilm Development

The effects of *A. eupatoria* extracts on biofilm formation and initial cell attachment were tested in 96‐well flat‐bottomed polystyrene TC‐treated microtiter plates, following a crystal violet staining assay as previously described by Stepanović et al. [30]. Twofold serial dilutions of plant extracts in a decreasing concentration range (10 mg/mL) were prepared in tryptic soy broth (TSB) (Torlak, Belgrade, Serbia) supplemented to a final glucose concentration of 1%. Afterward, 10 *μ*L of fresh bacterial suspension (0.5 McFarland turbidity standard) was added into the respective wells containing 100 *μ*L of sample per well. After inoculation, microtiter plates were incubated at 37°C for 4 h for the cell attachment inhibition assay, whereas the incubation for the biofilm formation inhibition assay lasted 20 h. After the incubation period, the content of each well was gently removed by pipetting, and wells were washed with sterile distilled water two times to remove nonadherent bacteria. In order to fix the formed biofilms, the microtiter plates were exposed to hot air at 55°C for 15 min. Then, the biofilms were stained with 100 *μ*L of an aqueous solution of crystal violet (0.1% w/v) (Fisher Scientific, Geel, Belgium). Following a 15‐min incubation period at room temperature, the excess stain was rinsed off by pipetting with distilled water three times. Then, the microtiter plates were dried in an inverted position, and 100 *μ*L of 10% acetic acid was added to release the dye bound to the cells. Gentle pipetting was done to mix the content properly. The samples′ optical densities (ODs) were measured at 550 nm using a microplate reader (RT‐2100C, Rayto, Shenzhen, China). The plant extracts were dissolved in 10% DMSO. Each experiment included growth control (broth + bacterium), extract control (broth + extract), broth control (broth only), and solvent control. The percentage of inhibition was calculated using the following Equation (2) presented by Ali et al. [31]:

(2) %of inhibition=ODGC−ODB−ODS−ODECODGC−ODB×100.

where OD_GC_ is the OD value of the growth control, OD_B_ is the OD value of the broth control, OD_s_ is the OD value of the sample, and OD_EC_ is the OD value of the extract control.

#### 2.4.2. Reduction of Pre‐Formed Biofilm

To investigate the potential effects of *A. eupatoria* extracts on preformed biofilms, 180 *μ*L of nutrient broth (TSB supplemented to a final glucose concentration of 1%) was first inoculated with 20 *μ*L of a bacterial suspension (0.5 McFarland turbidity standard) for each strain. The inoculated 96‐well flat‐bottomed polystyrene TC‐treated microtiter plates were then incubated at 37°C for 20 h without the extracts. After the incubation period, nonadherent bacteria were removed, and the wells were rinsed. Subsequently, 100 *μ*L of plant extracts at different doses (20, 10, 5, and 2.5 mg/mL) were added to the preformed biofilms, followed by an additional 20‐h incubation. Controls were prepared as described above. Biofilm biomass was quantified using the crystal violet staining assay, as previously described. The results were presented as a percentage reduction in biofilm biomass using Equation (2).

#### 2.4.3. Effect on Metabolic Activity of Biofilm

Resazurin dye was used to assess the impact of *A. eupatoria* extracts on biofilm metabolic activity [32]. Nutrient broth (TSB supplemented to a final glucose concentration of 1%), bacterial suspensions, and twofold dilutions of plant extracts (10 mg/mL) were prepared in 96‐well flat‐bottomed TC‐treated microtiter plates in the same manner as previously described. Also, a growth control (broth + bacterium), an extract control (broth + extract), and a solvent control were included. Following a 20‐h incubation period at 37°C, the content of each well was carefully removed, and the wells were washed with sterile distilled water two or three times. Then, 100 *μ*L of sterile nutrient broth and 10 *μ*L of an aqueous resazurin solution (0.05% w/v) were added to the wells, and the plates were incubated for an additional 2 h at 37°C. After incubation, the lowest concentrations of tested plant extracts that prevented a color change of the resazurin from blue to pink were visually determined and defined as biofilm metabolic inhibitory concentrations (BMICs).

#### 2.4.4. Effect on Auto‐Aggregation and Cell Surface Hydrophobicity of Bacteria

In both the absence and presence of *A. eupatoria* extracts, the auto‐aggregation behavior and cell surface hydrophobicity of *S. aureus* (S1 and S2), *Proteus* spp. (Pr1), and *P. aeruginosa* (PA2 and PA3) were investigated [33]. Shortly, 1 mL of bacterial suspension (2.0 McFarland turbidity standard) in phosphate‐buffered saline (PBS) (Fisher Scientific, Geel, Belgium) with 1 mL of tested plant extract (final concentration: 1 mg/mL, in 1% DMSO in PBS) was incubated at 37°C for 1 h. Following 10 min of centrifugation at 5000 rpm, the supernatant was discarded, and the pellet was washed and resuspended in 5 mL of PBS. In the auto‐aggregation assay, each sample′s OD was measured at 600 nm immediately (0 h of incubation) after 30 s of vortexing (OD_0_) and after 4 h of incubation at 37°C (OD_1_). To determine the effect on cell surface hydrophobicity, the obtained suspension was mixed with 1 mL of *p*‐xylene (Sigma‐Aldrich, St. Louis, United States), then vigorously vortexed for 30 s, and incubated for 30 min. Each sample′s aqueous phase′s OD was measured before and after adding *p*‐xylene (OD_0_ and OD_1_, respectively). The percentage of auto‐aggregation and hydrophobicity was calculated according to Equation (3):

(3) %=OD0−OD1/OD0×100

#### 2.4.5. Effect on Bacterial Motility

Using semisolid agar supplemented with or without *A. eupatoria* extracts in 6‐well microtiter plates, the swimming and swarming motility of *Proteus* spp. (Pr1) and *P. aeruginosa* (PA1, PA2, PA3, and PA4) were assessed. The final extract concentration was 1 mg/mL (1% DMSO final in PBS). For swimming motility, 0.3% agar containing 10 g/L of tryptone and 5 g/L of NaCl was used. LB medium supplemented with 5 g/L of glucose and 7 g/L of agar was utilized for swarming motility.

To inoculate media plates, 1 *μ*L of bacterial suspension (0.5 McFarland turbidity standard) was stabbed into the middle of the agar for swimming motility, and 1 *μ*L of suspension was added to the agar surface for swarming motility [34]. After incubation at 37°C for 20 h, the zone diameters (mm) of bacterial migration from the point of inoculation were measured and compared with the control.

#### 2.4.6. Fluorescence Microscopy

The impact of plant extracts on biofilm formation was observed using fluorescence microscopy (NIKON inverted fluorescence microscope, Ti‐Eclipse). *S*. *aureus* S2 strain was used as a model strain. A bacterial suspension adjusted to a 0.5 McFarland turbidity standard was treated with various extracts’ concentrations (ranging from 0.312 mg/mL to 10 mg/mL). Following 20 hours of incubation at 37°C, the biofilms were fixed and stained with 10 *μ*L of an aqueous solution of acridine orange (0.05% w/v) (Fisher Scientific, Geel, Belgium) for 1 min in the dark and then visualized at 400 × magnification. Acridine orange stains both live and dead cells in the biofilm and also binds to nucleic acids present in the extracellular matrix [35].

### 2.5. Statistical Analysis

For the statistical analysis, IBM SPSS Statistics for Windows, Version 21.0 (IBM Corp., Armonk, New York, United States), was used. Pairwise comparisons were evaluated using the Student′s *t*‐test, whereas differences among multiple groups were analyzed using one‐way analysis of variance (ANOVA), followed by Tukey′s post hoc test. Results are expressed as mean ± standard deviation (SD), and differences were considered statistically significant at *p* < 0.05.

## 3. Results

### 3.1. FT‐IR Spectroscopy

FT‐IR spectroscopy was used to characterize the structural features of constituents present in the *A. eupatoria* extracts. The spectra were interpreted based on the characteristic vibrational regions of phenolic compounds (Figures S1, S2, and S3) and comparison with reference FT‐IR spectra of representative phenolic standards (Figures S4, S5, and S6) recorded under identical conditions [22, 36–38]. The results are summarized in Table 1.

**Table 1:** FT‐IR absorption bands observed in *A. eupatoria* extracts.

**No.**	**Ethanol extract (cm** ^**−1**^ **)**	**Acetone extract (cm** ^**−1**^ **)**	**Ethyl acetate extract (cm** ^**−1**^ **)**	**Expected region (cm** ^**−1**^ **)**	**Functional group/assignment**	**Phytochemical class indicated**	**Referencestandard(s) showing analogous bands** ^**a**^
1	3426.79	3428.15	3427.54	3600–3200 (broad)	*ν*(O–H), hydrogen‐bonded	Polyphenols, flavonoids and hydrolysable tannins	Gallic, caffeic, and ferulic acids, quercetin·H₂, rutin, hesperidin
2	2923.92	2921.23	2922.25	2940–2915	*ν*(C–H) aliphatic (CH₂)	Glycosidic residues and polysaccharides	Rutin, hesperidin
3	2853.41	2850.68	2851.73	2860–2845	*ν*(C–H) aliphatic (CH₂)	Sugar moieties in glycosides	Rutin and hesperidin
4	/	1737.88	1734.52	1745–1725	*ν*(C=O) (ester/carboxyl)	Phenolic acids and esterified flavonoids	Gallic, syringic, and ferulic acids
5	1689.83	1690.25	1691.32	1700–1650	*ν*(C=O) (conjugated carbonyl)	Phenolic acids	Gallic, caffeic, ferulic, and protocatechuic acids
6	1618.78	1615.29	1616.49	1625–1600	Aromatic *ν*(C=C)	Flavonoid aglycones and phenolic cores	Quercetin·H₂O, rutin, and hesperidin
7	1516.73	1515.77	/	1525–1500	Aromatic *ν*(C=C) skeletal	Flavonoid aglycones	Quercetin·H₂O
8	1450.79	1461.87	1461.62	1470–1440	*δ*(C–H) bending	Aromatic substitution	All phenolic standards
9	1378.73	1376.96	1376.90	1390–1360	*δ*(C–H) bending	Polyphenolic rings	All phenolic standards
10	1279.70	1274.10	1274.15	1290–1260	*ν*(C–O) aromatic ether	Flavonoid glycosides and phenolic esters	Rutin, hesperidin, and quercetin·H₂O
11	/	1239.93	1239.55	1250–1220	*ν*(C–O) phenolic/aryl–O	Flavonoid ether linkages	Rutin and hesperidin
12	/	1199.84	/	1210–1180	*ν*(C–O)/*ν*(C–N) (mixed)	Glycosidic linkages	Hesperidin and rutin
13	/	1168.82	1166.50	1180–1155	*ν*(C–O) ring mode	Hydrolysable tannins and phenolic glycosides	Gallic acid
14	/	1105.85	1110.48	1120–1090	*ν*(C–O–C) (glycosidic)	Flavonoid glycosides and polysaccharides	Rutin and hesperidin
15	/	1075.90	1076.00	1085–1060	*ν*(C–O)/*ν*(C–N)	Glycosidic networks	Rutin and hesperidin
16	1057.09	1053.24	1051.06	1060–1045	*ν*(C–O) in aromatic system	Phenolic ring systems	Gallica and caffeic acids
17	/	/	1031.62	1040–1025	*ν*(C–O)	Glycosylated flavonoids	Rutin and hesperidin
18	/	/	998.37	1010–990	*δ*(C–H) (aromatic)	General phenolic matrix	All phenolic standards
19	866.01	865.70	866.19	875–860	*δ*(C–H) out‐of‐plane	Aromatic ring substitution	All phenolic standards
20	818.94	818.59	828.54	835–815	*δ*(C–H) out‐of‐plane	Aromatic ring substitution	All phenolic standards

^a^ Reference standards (hesperidin, rutin, quercetin·H₂O, gallic acid, syringic acid, caffeic acid, *trans*‐ferulic acid, and protocatechuic acid) were recorded under identical FT‐IR conditions and used only to illustrate characteristic vibrational regions; no compound‐level identification is inferred.

All three extracts exhibited a broad absorption band at ~3425 cm^−1^, attributed to hydrogen‐bonded O–H stretching vibrations [37, 39], which is characteristic of polyphenols, flavonoids, and hydrolysable tannins. Signals observed in the 2925–2850 cm^−1^ region correspond to aliphatic *ν*(C–H) stretching, indicating the presence of glycosidic and polysaccharide residues. The bands at ~1735–1685 cm^−1^ are assigned to *ν*(C=O) stretching modes associated with phenolic acids and esterified flavonoids, whereas the aromatic *ν*(C=C) skeletal vibrations at ~1618–1515 cm^−1^ support the presence of flavonoid aglycones and phenylpropanoid ring systems.

The region between 1290 and 1000 cm^−1^ showed prominent *ν*(C–O) and *ν*(C–O–C) stretching bands, particularly in the acetone and ethyl acetate extracts, indicating contributions from glycosidically linked flavonoids and hydrolysable tannins. Bands below 1000 cm^−1^ correspond to *δ*(C–H) out‐of‐plane deformations typical of substituted aromatic rings and represent the fingerprint region of phenolic structures. Notably, the *ν*(C=O) band was slightly more pronounced in the ethyl acetate extract, consistent with a higher relative contribution of phenolic acids and esterified flavonoids, whereas the broader signal in the ethanol extract reflects a greater proportion of glycosylated flavonoids and hydrolysable tannins.

### 3.2. Phytochemical Analysis

Among the groups of phenolic compounds analyzed in the extracts of *A. eupatoria*, the concentration of TPAs was the highest, followed by the concentrations of total flavonoids and total proanthocyanidins (Table 2). The ethyl acetate extract showed the highest measured concentration of TPAs (149.65 ± 0.73 mg CAE/g), followed by the ethanol and acetone extracts. In addition, the ethanol extract had the highest concentration of total flavonoids (89.87 ± 0.42 mg RUE/g) and total proanthocyanidins (58.97 ± 0.22 mg CchE/g), followed by acetone and ethyl acetate extracts, in both cases. Statistically significant differences were found in the contents among the groups of phenolic compounds (Table 2).

**Table 2:** Total phenolic compound content (TPC), total phenolic acid (TPA) content, total flavonoid content (TFC), and total proanthocyanidin content (TPAC) in *A. eupatoria* extracts.

**Type of extract**	**TPC (mg GAE/g)**	**TPA (mg CAE/g)**	**TFC (mg RUE/g)**	**TPAC (mg CchE/g)**
Ethanol	102.41 ± 0.25	130.60 ± 0.27	89.87 ± 0.42	58.97 ± 0.22
Acetone	66.07 ± 0.13^∗∗∗^^a^	113.94 ± 0.27^∗∗∗^^a^	81.68 ± 1.73^∗∗^^a^	26.8 ± 0.38^∗∗∗^^a^
Ethyl acetate	32.70 ± 0.19^∗∗∗^^a,^^∗∗∗^^b^	149.65 ± 0.73^∗∗∗^^a,^^∗∗∗^^b^	77.51 ± 2.14^∗∗∗^^a,^^∗^^b^	7.96 ± 0.18^∗∗∗^^a,^^∗∗∗^^b^

*Note:* Values are expressed as mean ± SD (*n* = 3). Statistical analysis was performed using one‐way ANOVA followed by Tukey′s post hoc test.

^a^ statistically significant difference in comparison with the ethanol extract.

^b^ statistically significant difference in comparison with the acetone extract.

∗*p* < 0.05.

∗∗*p* < 0.01.

∗∗∗*p* < 0.001.

### 3.3. Chemometric Analysis

PCA successfully discriminated the three extracts based on their FT‐IR spectral profiles. In the full spectrum analysis (4000–450 cm^−1^) of extracts alone (Figure S7), the first two principal components explained approximately 99% of the total variance (PC1: 77%, PC2: 22%). The PC1 scores demonstrated a clear gradient: ethanol extract exhibited the most negative score, acetone extract an intermediate position, and ethyl acetate extract the most positive score. The fingerprint region analysis (1800–650 cm^−1^) yielded comparable discrimination (PC1: 86%, PC2: 13%).

When authentic phenolic standards were included (Figure S8), the PCA score plot revealed distinct clustering patterns: all three extracts were positioned in the negative PC1 region. Ethanol and acetone extracts clustered in close proximity to flavonoid glycosides (rutin and hesperidin), whereas ethyl acetate extract trended toward phenolic acid standards. Hydroxycinnamic acids (caffeic and *trans*‐ferulic acids) occupied distinctly separate positions with highly positive PC1 scores, forming an isolated cluster distant from all extracts.

HCA using Ward′s method corroborated the PCA findings. In the extracts‐only analysis (Figure S9), ethyl acetate extract formed a separate branch, whereas ethanol and acetone extracts clustered together. When standards were included (Figure S10), the dendrogram revealed three major clusters: a polar cluster containing ethanol extract, acetone extract, rutin, and hesperidin; a phenolic acid cluster with ethyl acetate extract showing intermediate association; and a distinctly separate hydroxycinnamic acid cluster.

### 3.4. Minimum Inhibitory Concentration of *A. eupatoria* Extracts

To obtain the initial biofilm treatment concentrations of *A. eupatoria* extracts, the MICs of ethanol, acetone, and ethyl acetate extracts were determined and are presented in Table 3. The most effective extracts were acetone and ethyl acetate extract against the gram‐positive bacterium *S. aureus* at the highest tested dose of 10 mg/mL. Regarding the gram‐negative bacteria, ethanol and ethyl acetate extracts were inactive. Only the acetone extract was successful at 10 mg/mL against three strains of *P. aeruginosa*. The antibiotic tetracycline was active at concentrations ranging from 0.0005 mg/mL to 0.128 mg/mL. The solvent control did not inhibit bacterial growth.

**Table 3:** Minimum inhibitory concentration (MIC) (mg/mL) and biofilm metabolic inhibitory concentration (BMIC) (mg/mL) of *A. eupatoria* extracts and an antibiotic tetracycline (mg/mL).

**Bacterial strains**	**Ethanol extract**		**Acetone extract**		**Ethyl acetate extract**		**Tetracycline**
	**MIC**	**BMIC**	**MIC**	**BMIC**	**MIC**	**BMIC**	**MIC**
*S. aureus* S1	ND	ND	10	1.25	10	2.5	0.064
*S. aureus* S2	10	ND	10	ND	10	2.5	0.0005
*S. aureus* S3	ND	ND	10	5	10	5	0.016
*S. aureus* S4	ND	ND	10	10	10	10	0.032
*S. aureus* ATCC 25923	ND	ND	10	ND	ND	10	0.064
*Proteus* spp. Pr1	ND	ND	ND	ND	ND	ND	ND
*P. aeruginosa* PA1	ND	ND	10	ND	ND	ND	0.128
*P. aeruginosa* PA2	ND	ND	ND	ND	ND	10	0.128
*P. aeruginosa* PA3	ND	ND	10	ND	ND	ND	0.064
*P. aeruginosa* PA4	ND	ND	ND	ND	ND	ND	ND
*P. aeruginosa* ATCC 10145	ND	ND	10	ND	ND	ND	0.032

Abbreviation: ND, no inhibitory effect observed.

### 3.5. Antibiofilm Activity of *A. eupatoria* Extracts

#### 3.5.1. Inhibition of Biofilm Formation

**Figure 1:**
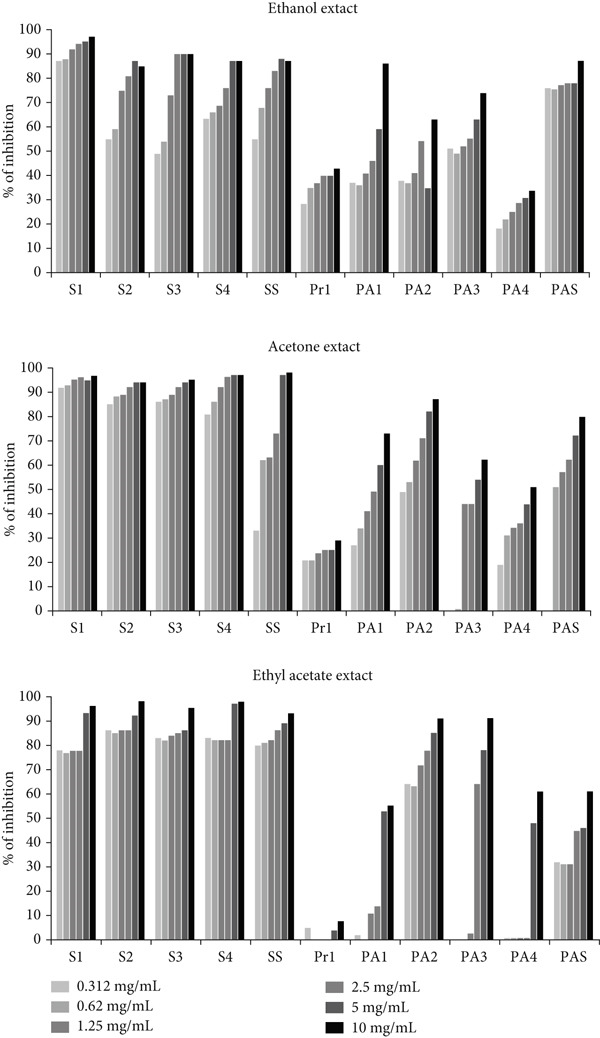
Inhibition of biofilm formation by different concentrations of *A. eupatoria* extracts (S1, S2, S3, S4—*S. aureus* isolates; SS—*S. aureus* ATCC 25923; Pr1*—Proteus* spp.; PA1, PA2, PA3, PA4—*P. aeruginosa* isolates; and PAS—*P. aeruginosa* ATCC 10145).

As shown in Figure 1, the effect of *A. eupatoria* extracts was both strain‐dependent and dose‐dependent. All extracts demonstrated a statistically significant suppression of biofilm formation in gram‐positive bacteria compared to gram‐negative bacteria (*p* < 0.05).

The acetone and ethyl acetate extracts inhibited biofilm formation of the tested gram‐positive strains by ≥ 75% at all tested concentrations, except for *S. aureus* ATCC 25923 in the presence of the acetone extract (33%). The ethanol extract inhibited biofilm formation by ≥ 45% in all tested gram‐positive strains at all tested concentrations. Regarding the tested gram‐negative strains, the extracts revealed better activity against *P. aeruginosa* strains than against *Proteus* spp. The ethanol extract inhibited biofilm formation of the tested gram‐negative strains by ≥ 22% at all tested concentrations. Further, the acetone extract inhibited biofilm formation by ≥ 19% in all tested gram‐negative strains, except for *P. aeruginosa* (PA3 and PAS). The ethyl acetate extract was the most active against *P. aeruginosa* (PA2 and PAS). The solvent control did not inhibit biofilm formation.

#### 3.5.2. Inhibition of Cell Attachment

**Figure 2:**
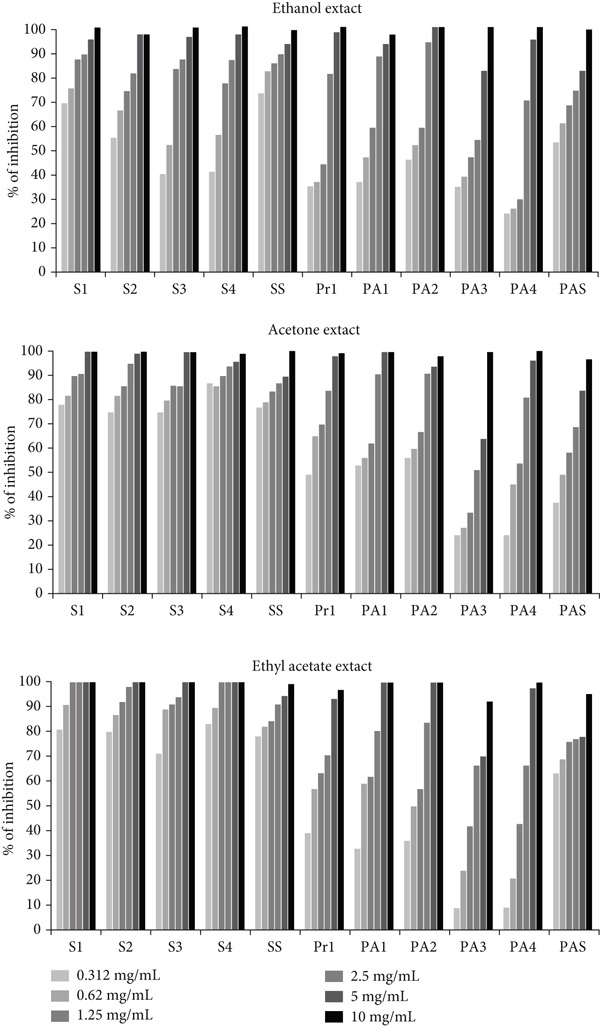
Inhibition of cell attachment by different concentrations of *A. eupatoria* extracts (S1, S2, S3, S4—*S. aureus* isolates; SS—*S. aureus* ATCC 25923; Pr1*—Proteus* spp.; PA1, PA2, PA3, PA4—*P. aeruginosa* isolates; and PAS*—P. aeruginosa* ATCC 10145).

The ethanol, acetone, and ethyl acetate extracts of *A. eupatoria* had a significant impact on the initial phase of biofilm formation. Compared with gram‐negative bacteria, gram‐positive bacteria exhibited a greater sensitivity to the examined extracts (*p* < 0.05). Although the acetone and ethyl acetate extracts prevented *S. aureus* cell attachment by ≥ 70% at all tested concentrations, the ethanol extract prevented *S. aureus* cell attachment by ≥ 40% at all tested concentrations (Figure 2). The tested extracts also prevented the development of biofilms of gram‐negative bacteria in a high percentage. The acetone, ethanol, and ethyl acetate extracts prevented cell attachment of gram‐negative strains at all tested concentrations by ≥ 37%, 35%, and 33%, respectively, except for two *P. aeruginosa* strains (PA3 and PA4). The solvent control had no negative impact on the bacteria.

#### 3.5.3. Inhibition of Preformed Biofilm

**Figure 3:**
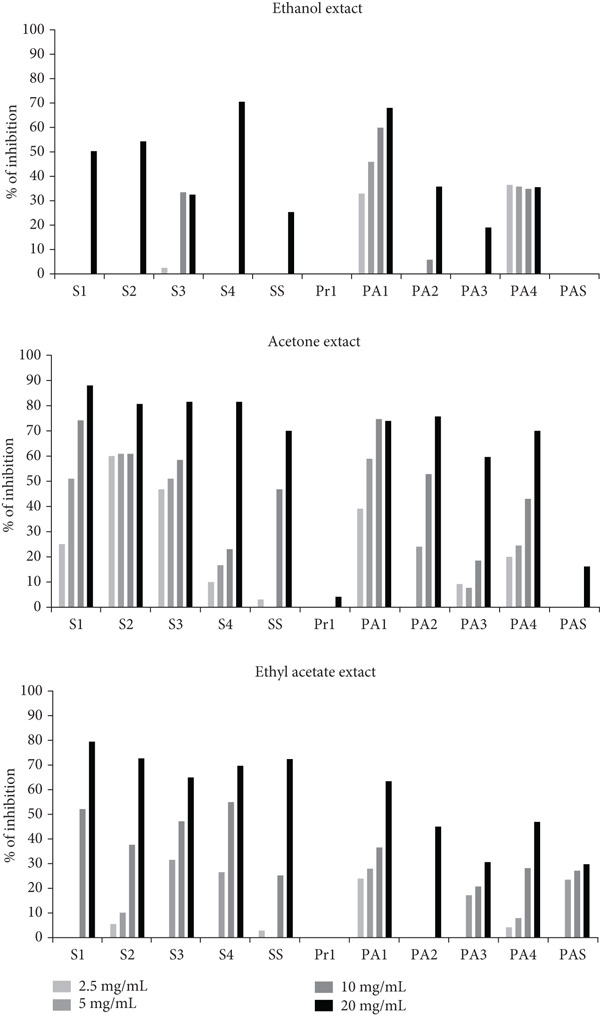
Reduction of preformed biofilm by different concentrations of *A. eupatoria* extracts (S1, S2, S3, S4—*S. aureus* isolates; SS—*S. aureus* ATCC 25923; Pr1*—Proteus* spp.; PA1, PA2, PA3; PA4—*P. aeruginosa* isolates; and PAS*—P. aeruginosa* ATCC 10145).

Among the tested extracts, the acetone extract showed the most remarkable ability to reduce the preformed biofilm of the tested strains (Figure 3). The effects of the ethyl acetate and ethanol extracts on the reduction of the established biofilms were weaker or absent (Figure 3). Preformed biofilms of *S. aureus* strains were the most susceptible (inhibition up to 88%), followed by strains of *P. aeruginosa* (inhibition up to 76%). The extracts were inactive against preformed biofilm of *Proteus* spp. The solvent control had no negative impact on the bacteria.

#### 3.5.4. Effect on Metabolic Activity of Biofilm

The acetone and ethyl acetate extracts inhibited the viability of gram‐positive strains at concentrations ranging from 1.25 mg/mL to 10 mg/mL (Table 3). The acetone extract showed a better inhibitory effect on the viability of *S. aureus* S1 biofilm than the ethyl acetate extract, whereas for *S. aureus* S2, the inhibitory effect of the ethyl acetate extract on biofilm viability was greater. Additionally, the metabolic inhibitory effect of the ethyl acetate extract on the biofilm of *P. aeruginosa* PA2 was observed at a concentration of 10 mg/mL (Table 3). The ethanol extract did not inhibit the biofilm viability of the tested strains, even at a concentration of 10 mg/mL. The solvent control was not toxic to bacteria.

#### 3.5.5. Effect on Auto‐Aggregation and Cell Surface Hydrophobicity

Table 4 displays the range of auto‐aggregation for the investigated bacterial strains, from 8.20*%* ± 2.99*%* to 36.48*%* ± 1.61*%*. In the presence of ethanol, acetone, and ethyl acetate extracts of *A. eupatoria*, the auto‐aggregation ability of all tested strains increased.

**Table 4:** Effect of *A. eupatoria* extracts on auto‐aggregation.

**Bacterial strains**	**Control (%)**	**Ethanol extract (%)**	**Acetone extract (%)**	**Ethyl acetate extract (%)**
*S. aureus* S1	17.58 ± 0.75	26.50 ± 4.46^∗^	48.02 ± 0.08^∗∗∗^	52.96 ± 0.19^∗∗∗^
*S. aureus* S2	12.66 ± 1.18	31.67 ± 1.32^∗∗^	47.35 ± 5.30^∗∗^	48.07 ± 2.58^∗∗^
*Proteus* spp. Pr1	8.20 ± 2.99	78.65 ± 0.10^∗∗^	30.09 ± 3.28^∗∗^	87.38 ± 0.73^∗∗∗^
*P. aeruginosa* PA2	36.48 ± 1.61	51.91 ± 0.73^∗∗^	70.07 ± 0.32^∗∗^	53.38 ± 4.38^∗^
*P. aeruginosa* PA3	13.67 ± 1.55	63.73 ± 2.49^∗∗^	75.17 ± 2.26^∗∗^	79.94 ± 0.38^∗∗∗^

*Note:* Values are expressed as mean ± SD (*n* = 3). Statistical analysis was performed using Student′s *t*‐test.

∗*p* < 0.05, ∗∗*p* < 0.01, and ∗∗∗*p* < 0.001, asterisks indicate significant differences compared with control.

The hydrophobicity of the cell surface toward *p*‐xylene varied between 11.95*%* ± 8.80*%* and 59.52*%* ± 14.81*%*. Nevertheless, the examined *A. eupatoria* extracts did not decrease cell surface hydrophobicity (data not shown).

#### 3.5.6. Effect on Bacterial Motility

The motility zones of all studied strains ranged from 21 mm to 37 mm, indicating swimming movement. However, only two strains, *Proteus* spp. (Pr1) and *P. aeruginosa* (PA1), displayed swarming motility (with motility zones measuring 30 and 35 mm, respectively).

The swimming motility zone of *P. aeruginosa* PA4 was reduced by the ethanol, acetone, and ethyl acetate extracts of *A. eupatoria* (Table 5, Figure S11). Additionally, only the acetone extract decreased swimming motility zones of *P. aeruginosa* ATCC 10145. The results showed that the tested extracts did not affect the swimming motility of *Proteus* spp. (Pr1) or *P. aeruginosa* (PA1, PA2, and PA3).

**Table 5:** Effect of *A. eupatoria* extracts on swimming motility.

**Bacterial strains**	**Control**	**Ethanol extract**	**Acetone extract**	**Ethyl acetate extract**
	**Swimming motility zone (mm)**			
*Proteus* spp. Pr1	37.07 ± 0.25	37.07 ± 0.38	37.10 ± 0.26	37.00 ± 0.26
*P. aeruginosa* PA1	36.93 ± 0.31	37.03 ± 0.31	37.17 ± 0.25	37.07 ± 0.25
*P. aeruginosa* PA2	37.13 ± 0.31	37.03 ± 031	37.03 ± 0.15	37.07 ± 0.25
*P. aeruginosa* PA3	21.17 ± 0.35	31.07 ± 0.6^∗∗∗^	30.33 ± 0.60^∗∗∗^	23.07 ± 0.35^∗∗^
*P. aeruginosa* PA4	27.03 ± 0.31	23.03 ± 0.70^∗∗^	19.17 ± 0.7^∗∗∗^	24.97 ± 0.35^∗∗^
*P. aeruginosa* ATCC 10145	26.12 ± 0.35	30.13 ± 0.70^∗∗^	21.93 ± 0.60^∗∗∗^	28.10 ± 0.66^∗^

*Note:* Values are expressed as mean ± SD (*n* = 3). Statistical analysis was performed using Student′s *t*‐test.

∗*p* < 0.05, ∗∗*p* < 0.01, and ∗∗∗*p* < 0.001, asterisks indicate significant differences compared with control.

Regarding the swarming motility, the ethanol extract reduced the swarming motility of *Proteus* spp. (Pr1), whereas the ethyl acetate extract decreased the swarming motility of *P. aeruginosa* (PA1) (Table 6, Figure S12).

**Table 6:** Effect of *A. eupatoria* extracts on swarming motility.

**Bacterial strains**	**Control**	**Ethanol extract**	**Acetone extract**	**Ethyl acetate extract**
	**Swarming motility zone (mm)**			
*Proteus* spp. Pr1	30.03 ± 0.32	19.03 ± 0.31^∗∗∗^	37.03 ± 0.40^∗∗∗^	37.17 ± 0.35^∗∗∗^
*P. aeruginosa* PA1	35.00 ± 0.30	37.17 ± 0.35^∗∗^	37.13 ± 0.25^∗∗^	27.03 ± 0.60^∗∗∗^

*Note:* Values are expressed as mean ± SD (*n* = 3). Statistical analysis was performed using Student′s *t*‐test.

∗*p* < 0.05, ∗∗*p* < 0.01, and ∗∗∗*p* < 0.001, asterisks indicate significant differences compared with control.

#### 3.5.7. Fluorescence Microscopy

**Figure 4:**
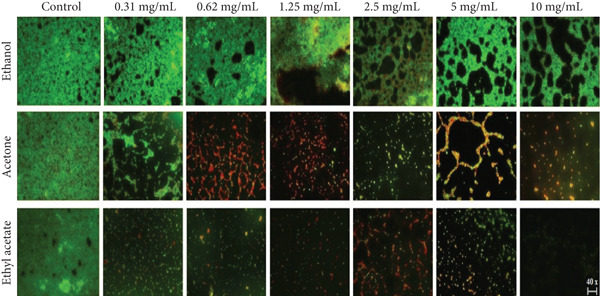
Fluorescence microscope images of *S. aureus* biofilm after treatment with *A. eupatoria* extracts.

The effect of *A. eupatoria* extracts on biofilm formation was confirmed by microscopic visualization using a fluorescence microscope. As observed in the control samples, *S. aureus* (S2) formed a dense and robust biofilm. As expected, the biofilm‐forming capacity gradually decreased with increasing extract concentration (Figure 4). The OD values were consistent with a decrease in the density of adhering bacterial cells. As confirmed by the biofilm inhibition assay, the acetone and ethyl acetate extracts were more active than the ethanol extract, which was visualized by the reduction in biofilm biomass.

## 4. Discussion

Skin and wound infections are among the most common health problems affecting people of all ages. In addition to standard pharmacotherapy, medicinal plants and their constituents are often employed as complementary therapeutic options. Experimental studies have increasingly investigated the effects and mechanisms of natural plant products, revealing complex actions that may support the management of skin and wound infections [40]. These infections are often associated with biofilms, which contribute to delayed wound healing and complicate treatment outcomes [41]. The high prevalence and resilience of biofilm‐related infections have driven the search for novel strategies, including plant‐derived agents, whose bioactive compounds may disrupt bacterial colonization and persistence. In this study, various extracts of the plant *A. eupatoria* were therefore investigated as a potential agent for biofilm control. *A. eupatoria* has attracted scientific interest due to its extensive use in traditional medicine, particularly in European and Asian ethnomedicine, where aerial parts in the flowering stage are used [14]. Despite this, scientific evidence regarding its antibiofilm properties and underlying mechanisms remains limited, emphasizing the need for further systematic investigation.

Previous studies examining the phytochemical composition of different extracts of *A. eupatoria* agree on the main groups of secondary metabolites, namely tannins, phenolic acids, and flavonoids; as well as primary metabolites; monosaccharides; and amino acids. Huzio et al. [42] investigated phytochemical and pharmacological aspects of *A. eupatoria* ethanol extract, identifying a total of 11 free and 17 bound monosaccharides, as well as 17 amino acids, including nine essential ones. Using the HPLC analysis, they detected gallic and ellagic acids, gallocatechin, epigallocatechin, catechin, epicatechin, and epicatechin gallate, along with hydroxycinnamic acids, such as hydroxyphenylacetate, caffeic acid, syringic acid, *p*‐coumaric acid, ferulic acid, sinapic acid, cinnamic acid and quinic acid; flavonoids quercetin‐3‐D‐glucoside (isoquercitrin), neohesperidin, naringenin and luteolin were found. Similar groups of compounds were identified in the extracts used in our research. Based on the bands observed in the FT‐IR spectra, it can be concluded that the examined extracts contain compounds from the group of phenols, phenolic acids, and flavonoids. The ethanol extract was the one with the highest content of most of the tested groups of compounds (phenols, flavonoids, and proanthocyanidins), with the exception of phenolic acids, which are the most abundant in the ethyl acetate extract. According to Balážová et al. [43], the TFC in *A. eupatoria* extracts decreased in the following order: methanolic (2.58 ± 0.7 mg QE/100 g DW), ethanolic (1.96 ± 0.3 mg QE/100 g DW), aqueous (1.51 ± 0.1 mg QE/100 g DW), and acetone extract (1.13 ± 0.5 mg QE/100 g DW). The methanol extract showed the highest content of TPA (9.71 ± 0.3 mg CGAE/100 g DW), the aqueous extract slightly less (9.15 ± 1.3 mg CGAE/100 g DW), and the acetone extract the lowest content of TPA (4.18 ± 1.0 mg CGAE/100 g DW). Further, the HPLC‐DAD analysis of aqueous extract revealed the presence of seven phenolic compounds, with rutin being the most abundant (6.56 mg/L), followed by *p*‐coumaric acid, *o*‐coumaric acid, caffeic acid, and chlorogenic acid [43]. In the study by Muruzović et al. [15], the highest concentration of total flavonoids (97.06 gRU/g), total phenols (220.31 mgGA/g), total tannins (207.27 mgGA/g), and total proanthocyanidins (103.72 CChE/g) was found in the *A. eupatoria* acetone extract. Taken together, these findings indicate that the extracts of *A. eupatoria* are particularly rich in phenolic compounds and flavonoid classes. The relative abundance of these compounds in tested extracts likely contributes to the pronounced antibiofilm effects observed in our study. However, since crude extracts represent complex mixtures of numerous bioactive and inactive components, the precise contribution of individual metabolites remains uncertain.

The multivariate analysis of FT‐IR spectra successfully differentiated the three extracts based on their phytochemical profiles, confirming that solvent polarity plays a crucial role in the selective extraction of bioactive compounds [44]. The PCA separation along PC1 reflects compositional differences: Ethanol extract exhibited a polar phenolic profile consistent with preferential extraction of phenolic acids and flavonoids; acetone extract showed intermediate composition reflecting effectiveness for both polar phenolics and semipolar terpenoids; and ethyl acetate extract trended toward phenolic acid standards, indicating enrichment in less polar phenolic compounds. The HCA clustering patterns corroborated these findings, positioning FT‐IR spectroscopy coupled with PCA and HCA as a valuable tool for quality control and standardization of plant extracts [45].

A particularly significant finding is the distinct separation of hydroxycinnamic acids (caffeic and *trans*‐ferulic acids) from all extracts in both PCA and HCA. The large Euclidean distances indicate these compounds are absent or present only in trace amounts in *A. eupatoria* aerial parts, distinguishing *Agrimonia* from many Rosaceae family plants where hydroxycinnamic acids constitute major phenolic components. Instead, the close association with flavonoid glycosides (rutin and hesperidin) and benzoic acid derivatives (gallic, protocatechuic, and syringic acids) suggests that *A. eupatoria* bioactive properties are primarily mediated by these compound classes. This finding is consistent with previous phytochemical studies identifying ellagitannins and flavonoid glycosides as predominant phenolic constituents of the *Agrimonia* species [42, 46].

The fingerprint region analysis (1800–650 cm^−1^) proved particularly informative for discriminating phenolic compound classes, encompassing characteristic vibrations of aromatic C=C stretching, C–O stretching, and aromatic C–H bending modes. The comparable discrimination achieved with this region alone suggests advantages for rapid screening applications by minimizing interference from less specific O–H and C–H stretching bands, with practical implications for developing robust chemometric models for *A. eupatoria* extract authentication and standardization.

The antibacterial and antibiofilm effect of plant extracts is related to their chemical composition. Compounds such as polyphenols (e.g., flavonoids, tannins, anthocyanins, phenolic acids, and coumarins) can interfere with bacterial cell processes including quorum sensing, enzyme activity, and cell wall synthesis, influence bacterial regulatory mechanisms and suppress bacterial virulence factors such as biofilm formation [47–49]. Therefore, the influence of *A. eupatoria* extracts on biofilm formation, the initial phase of biofilm formation, and the preformed biofilm were determined. All extracts significantly reduced biofilm formation in gram‐positive strains, with the acetone extract showing the strongest activity, followed by the ethyl acetate and ethanol extracts, highlighting how differences in chemical composition translate into distinct antibiofilm potency. These differences likely reflect variations in the phytochemical profiles obtained with different solvents, since solvent polarity determines the type and concentration of extracted secondary metabolites, which can influence their interaction with bacterial surfaces and the disruption of biofilm architecture [50]. Also, phenolic acids and flavonoids, which are previously identified as dominant constituents of the tested extracts, are known to interfere with biofilm‐related signaling and matrix production, which may account for the stronger effect of the more phenolic‐rich extracts [50]. In contrast, gram‐negative bacteria are less susceptible to the effects of the extracts, likely because of the outer membrane rich in lipopolysaccharides that limits the penetration of hydrophobic compounds and the activity of efflux pumps that expel phenolic constituents from the cell. This structural barrier has been widely recognized as a major factor conferring inherent resistance of gram‐negative bacteria to plant‐derived antimicrobials, contextualizing the observed differences between gram‐positive and gram‐negative bacteria [51]. Consistent with these observations, our previous study also demonstrated stronger biofilm activity of the acetone extract of *A. eupatoria* than the ethanol extract [52].

We also examined the impact on biofilm viability. The acetone and ethyl acetate extracts exerted the strongest effects on metabolic activity, whereas the ethanol extract showed no inhibition of biofilm viability in the tested strains. These results suggest that certain extract constituents may preferentially target biofilm‐associated metabolic processes rather than planktonic growth. The results of our previous research showed that the acetone extract of *A. eupatoria* inhibited the biofilm viability of *E. coli* (E16) at the same concentration as the ethanol extract. Still, the ethanol extract had more potent activity on *P. aeruginosa* (PA8) than the acetone extract [52]. The study by Muruzović et al. [15] showed that water and acetone extracts of *A. eupatoria* prevent the biofilm formation of *P. aeruginosa* and *P. mirabilis*, with the acetone extract showing superior activity. In addition, all tested extracts significantly affected the initial phase of biofilm formation, reducing cell adhesion in both gram‐positive and gram‐negative strains, with acetone and ethyl acetate extracts exhibiting the strongest effect. The preformed biofilm was gradually damaged after exposure to *A. eupatoria* extracts, with the acetone extract causing the most pronounced structural damage in both gram‐positive and gram‐negative bacteria. To the best of our knowledge, this is the first study demonstrating that *A. eupatoria* extracts can interfere with both initial cell attachment and established biofilms, indicating activity at multiple stages of biofilm development.

The PCA results demonstrated that ethyl acetate extract exhibits greater spectral similarity to phenolic acid standards, consistent with its highest phenolic acid content (149.65 ± 0.73 mg CAE/g). Phenolic acids possess amphiphilic properties facilitating penetration through bacterial membranes and the hydrated EPS matrix. Phenolic acids can also chelate metal ions essential for biofilm stability, disrupt membrane integrity, and generate reactive oxygen species [53]. The enhanced ethyl acetate extract′s activity likely reflects this superior penetration combined with direct antimicrobial activity. The acetone extract′s intermediate PCA position, clustering with both flavonoid glycosides and phenolic acids, provides a chemical basis for its strongest antibiofilm activity. This dual extraction capacity creates synergistic effects where flavonoids contribute direct antibacterial activity while phenolic acids enhance penetration [54]. Flavonol glycosides like rutin can inhibit biofilm formation through antioxidant activity, direct binding to bacterial proteins, and modulation of adhesion [55]. In contrast, the ethanol extract, despite the highest flavonoid and TPAC, exhibited more moderate antibiofilm activity and no inhibition of biofilm metabolic viability. The PCA clustering with flavonoid glycosides explains this limitation. Glycosylated flavonoids are large polar molecules (> 600 Da) whose size and polarity impede penetration through lipophilic bacterial membranes and the dense EPS matrix [56]. This explains why ethanol extract effectively inhibited initial cell adhesion but was less effective against established biofilms.

Bacterial auto‐aggregation and cell hydrophobicity play an important role in biofilm formation through complementary physical and molecular mechanisms. During the initial steps of biofilm formation, along with colonization of inorganic surfaces or attachment to host cells, many bacteria have the ability to bind to themselves [57]. Furthermore, bacterial cell surface properties, including bacterial cell surface hydrophobicity and cell surface charge, strongly influence this attachment process [58, 59]. Therefore, if the auto‐aggregation or cell hydrophobicity decreases as a result of the treatment, biofilm formation is also likely to decrease. However, despite the observed antibiofilm effects, ethanol, acetone, and ethyl acetate extracts of *A. eupatoria* increased both auto‐aggregation and cell surface hydrophobicity in tested bacterial strains. Similar effects have been reported for the acetone extracts of *Ceratonia siliqua*, *Salix babylonica*, *Ziziphus mucronatai*, as well as methanol extracts of *Ehretia rigida*, *Leucaena leucocephala*, *Senegalia galpinii*, and *Vachellia sieberiana*, which promoted auto‐aggregation of *E. coli* O157:H7 [60]. Likewise, auto‐aggregation of *S. mutans* (ATCC 25175 and ATCC 35668), *S. salivarius* (ATCC 13419), and *S. mitis* (ATCC 49456) was significantly increased by the oolong tea extract, but the cell surface hydrophobicity was reduced [61]. In the study by Hui and Dykes [62], water extract of *Garcinia atroviridis* increased the hydrophobicity of *Bacillus cereus* ATCC 14579, *S. aureus* ATCC 25923, and *Salmonella enteritidis* ATCC 13076. Overall, increased cell hydrophobicity is often linked to changes in adhesion expression or surface appendages, although the precise mechanisms by which plant‐derived compounds induce these modifications remain unclear. Notably, the correlation between surface hydrophobicity, auto‐aggregation, and biofilm formation cannot be generalized, as it depends on bacterial species, environmental conditions, and the nature of the interacting surface [62].

For initial colonization and surface adhesion, particularly during the early stages of biofilm formation, swimming motility enables bacterial attachment, whereas swarming motility facilitates invasion and dissemination across surfaces [34]. Thus, disruption of motility may hinder the spread of infection and prevent biofilm formation. The results of our study showed that the acetone extract of *A. eupatoria* had the strongest inhibitory effect on swimming motility of the tested bacterial strains, followed by ethanol and ethyl acetate extracts. Regarding the swarming motility, only ethanol and ethyl acetate extracts of *A. eupatoria* reduced the motility of the tested bacterial strains, whereas the acetone extract had no significant effect. These findings suggest that *A. eupatoria* extracts may affect bacterial colonization and dissemination, not just through direct antimicrobial activity, but also by modulating behavioral traits crucial for biofilm formation. As far as we know, there are no previous studies on the effects of *A. eupatoria* extracts on bacterial motility. However, there are few studies on the effect of plant extracts from the genus *Agrimonia* and the Rosaceae family on bacterial motility. The effect of an ethanol extract of *A. pilosa* on swimming, swarming, and twitching motility of *P. aeruginosa* PAO1 was investigated [63]. The concentrations of the tested extract that reduced the motility of *P. aeruginosa* PAO1 were 0.28 and 5.6 mg/mL. The potential of *Rosa rugosa* tea polyphenol extract to inhibit swarm motility of *E. coli* K‐12 and *P. aeruginosa* PAO1 was concentration‐dependent; at a concentration of 640 *μ*g/mL, the motility inhibition of *E. coli* K‐12 and *P. aeruginosa* PAO1 was 84.90% and 78.03%, respectively [64]. Together, these data support the notion that certain phenolic‐rich plant extracts can interfere with motility‐related processes, although the specific molecular mechanisms underlying these effects remain to be elucidated.

## 5. Conclusions

According to the phytochemical analysis, *A. eupatoria* extracts were rich in phenolic compounds, particularly phenolic acids and flavonoids, which are known to modulate bacterial adhesion and biofilm‐related processes. In line with this, extracts of *A. eupatoria* exhibited significant antibiofilm activity against bacterial strains isolated from human wounds. Initial cell attachment and biofilm formation were significantly inhibited in the presence of *A. eupatoria* extracts, but the reduction of the established biofilm was less effective. The observed effects were strain‐dependent and dose‐dependent, with gram‐positive strains being more susceptible than the gram‐negative ones. Although the extracts did not reduce auto‐aggregation or cell hydrophobicity, swimming and swarming motilities were inhibited.

Overall, these results indicate that *A. eupatoria* may represent a promising source of phenolic‐rich and flavonoid‐rich compounds with antibiofilm potential. Although these findings are encouraging, the observed antibiofilm effects occurred at relatively high extract concentrations typical for crude plant preparations, and the study was limited to a defined set of bacterial isolates. Further studies should isolate and characterize the active molecules, elucidate their mechanisms of action, and assess their effects in more complex biological models before considering potential biomedical applications.

## Conflicts of Interest

The authors declare no conflicts of interest.

## Author Contributions

Conceptualization: J.N.T. and O.D.S. Formal analysis: J.N.T Investigation: J.N.T. Methodology: O.D.S and A.G.K Supervision: O.D.S. Validation: J.N.T., M.M.S., O.D.S., and A.G.K. Writing—original draft: J.N.T. and A.G.K. Writing—review and editing: M.M.S. and O.D.S.

## Funding

This work was funded by the Ministry of Science, Technical Development and Innovation of the Republic of Serbia, 451‐03‐137/2025‐03/200122 and COST Action CA23152 – Building Consensus on Biofilm Regulatory Decision Making (RegulatoryToolBox).

## Supporting information


**Supporting Information** Additional supporting information can be found online in the Supporting Information section. (Figures S1–S6) FT‐IR Spectroscopy. FT‐IR spectra of *A. eupatoria* ethanol, acetone, and ethyl acetate extracts (Figures S1–S3) were recorded to identify the major functional groups associated with phenolic and other bioactive constituents. To facilitate accurate band assignments, spectra of reference phenolic standards—hesperidin, rutin, quercetin‐hydrate, gallic acid, syringic acid, caffeic acid, *trans*‐ferulic acid, and protocatechuic acid are additionally presented (Figures S4–S6). Comparison of corresponding vibrational regions enabled more confident interpretation of characteristic peaks linked to flavonoids, hydroxycinnamic acids, and other phenolic components present in the extracts. (Figures S7–S10) Principal component analysis (PCA) and hierarchical cluster analysis (HCA). The chemical variability among the *A. eupatoria* extracts and their relationship with reference standards were explored using PCA and HCA analyses (Figures S7–S10). The PCA score plots (Figures S7–S8) illustrate the distribution and separation of vector‐normalized and autoscaled FT‐IR spectra of the acetone, ethyl acetate, and ethanol extracts, as well as their positioning relative to the selected analytical standards. The HCA dendrograms (Figures S9–S10), generated using Ward′s linkage and squared Euclidean distance, depict the clustering patterns within the three extracts and between the extracts and the eight reference standards, providing additional insight into their spectral similarity. (Figures S11–S12) Motility assays. The effects of *A. eupatoria* extracts on bacterial motility were evaluated using swimming and swarming assays. Representative photographs demonstrating the impact on swimming motility are provided in Figure S11 (Pr1—*Proteus* spp.; PA1–PA4—*P. aeruginosa* isolates; PAS—*P. aeruginosa* ATCC 10145). The impact of tested extracts on swarming motility is shown in Figure S12 (Pr1—*Proteus* spp.; PA1—*P. aeruginosa* isolate).

## Data Availability

The data that support the findings of this study are included in the article/Supporting Information. Further inquiries can be directed to the corresponding author.
