# Supplementary material for: Antibiofilm Activity of Agrimonia eupatoria Extracts Against Clinically Relevant Pathogens
Source: Int J Microbiol. 2026 Feb 20;2026:5222416. doi: 10.1155/ijm/5222416 (PMC12921527; doi:10.1155/ijm/5222416)
Supplement: Supplementary file 1 — Supporting Information Additional supporting information can be found online in the Supporting Information section. (Figures S1–S6) FT‐IR Spectroscopy. FT‐IR spectra of A. eupatoria ethanol, acetone, and ethyl acetate extracts (Figures S1–S3) were recorded to identify the major functional groups associated with phenolic and other bioactive constituents. To facilitate accurate band assignments, spectra of reference phenolic standards—hesperidin, rutin, quercetin‐hydrate, gallic acid, syringic acid, caffeic acid, trans‐ferulic acid, and protocatechuic acid are additionally presented (Figures S4–S6). Comparison of corresponding vibrational regions enabled more confident interpretation of characteristic peaks linked to flavonoids, hydroxycinnamic acids, and other phenolic components present in the extracts. (Figures S7–S10) Principal component analysis (PCA) and hierarchical cluster analysis (HCA). The chemical variability among the A. eupatoria extracts and their relationship with reference standards were explored using PCA and HCA analyses (Figures S7–S10). The PCA score plots (Figures S7–S8) illustrate the distribution and separation of vector‐normalized and autoscaled FT‐IR spectra of the acetone, ethyl acetate, and ethanol extracts, as well as their positioning relative to the selected analytical standards. The HCA dendrograms (Figures S9–S10), generated using Ward′s linkage and squared Euclidean distance, depict the clustering patterns within the three extracts and between the extracts and the eight reference standards, providing additional insight into their spectral similarity. (Figures S11–S12) Motility assays. The effects of A. eupatoria extracts on bacterial motility were evaluated using swimming and swarming assays. Representative photographs demonstrating the impact on swimming motility are provided in Figure S11 (Pr1—Proteus spp.; PA1–PA4—P. aeruginosa isolates; PAS—P. aeruginosa ATCC 10145). The impact of tested extracts on swarming motility is shown in F [file IJM-2026-5222416-s001.docx]

**Antibiofilm activity of *Agrimonia eupatoria* extracts against clinically relevant pathogens**

Jelena N. Terzić^1,2^, Marina M. Stanković^1^, Olgica D. Stefanović^1*^

^1^Department of Biology and Ecology, Faculty of Science, University of Kragujevac, Kragujevac, Serbia

^2^Department of Pharmacy, Faculty of Medical Science, University of Kragujevac, Kragujevac, Serbia

*Corresponding author: Olgica D. Stefanović, e-mail: [olgica.stefanovic@pmf.kg.ac.rs](mailto:olgica.stefanovic@pmf.kg.ac.rs)

**Captions**

**Fig. S1.** FT-IR spectrum of *A. eupatoria* ethanol extract.

**Fig. S2.** FT-IR spectrum of *A. eupatoria* acetone extract.

**Fig. S3.** FT-IR spectrum of *A. eupatoria* ethyl acetate extract.

**Fig. S4.** FT-IR spectra of flavonoid standards: A – hesperidin, B – rutin, C – quercetin hydrate.

**Fig. S5**. FT-IR spectra of hydroxybenzoic acid standards: A – gallic acid, B – syringic acid, C – protocatechuic acid.

**Fig. S6.** FT-IR spectra of hydroxycinnamic acid standards: A – caffeic acid, B – *trans*-ferulic acid.

**Fig. S7.** Principal Component Analysis (PCA) score plot of FT-IR spectra. PCA score plot of vector-normalized and autoscaled FT-IR spectra of acetone, ethyl acetate, and ethanol extracts. PC1 (77%) and PC2 (22%) capture all spectral variance, enabling clear separation of extracts based on solvent-dependent chemical composition.

**Fig. S8.** Principal Component Analysis (PCA) score plot of FT-IR spectra. PCA score plot of vector-normalized and autoscaled FT-IR spectra of extracts and standards. PC1 (45.4%) and PC2 (16%) capture all spectral variance, enabling clear separation of extracts based on solvent-dependent chemical composition.

**Fig. S9.** Hierarchical Cluster Analysis (HCA) dendrogram of FT-IR spectra. Dendrogram generated using Ward's linkage and squared Euclidean distance, illustrating clustering patterns among the three extracts. Acetone and ethyl acetate extracts cluster together, while the ethanol extract forms a separate branch, reflecting differences in their FT-IR spectral profiles.

**Fig. S10.** Hierarchical Cluster Analysis (HCA) dendrogram of FT-IR spectra. Dendrogram generated using Ward's linkage and squared Euclidean distance, illustrating clustering patterns among the three extracts and eight standards.

**Fig. S11.** Effect of *A. eupatoria* extracts on swimming motility of *Proteus* spp. (Pr1) and *Pseudomonas aeruginosa* isolates (PA1–PA4 and PAS).

**Fig. S12.** Effect of *A. eupatoria* extracts on swarming motility of *Proteus* spp. (Pr1) and *Pseudomonas aeruginosa* (PA1).

**
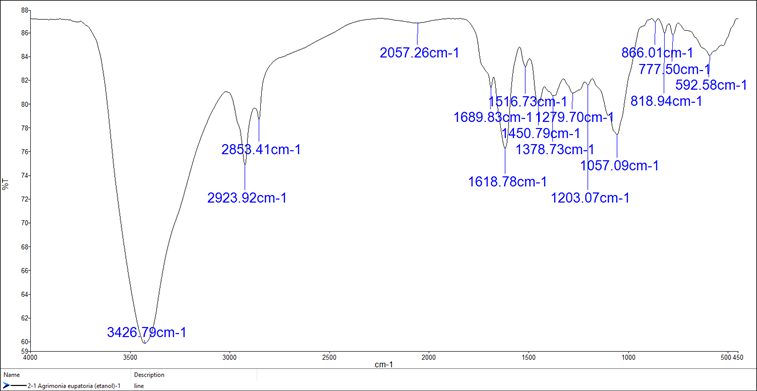
Fig. S1.** FT-IR spectrum of *A. eupatoria* ethanol extract.

**
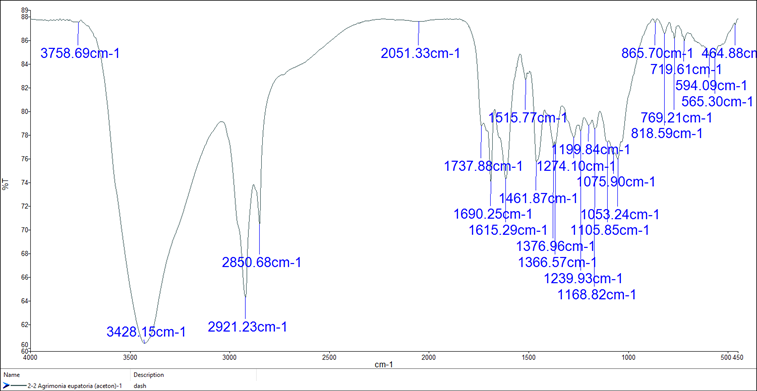
Fig. S2.** FT-IR spectrum of *A. eupatoria* acetone extract.


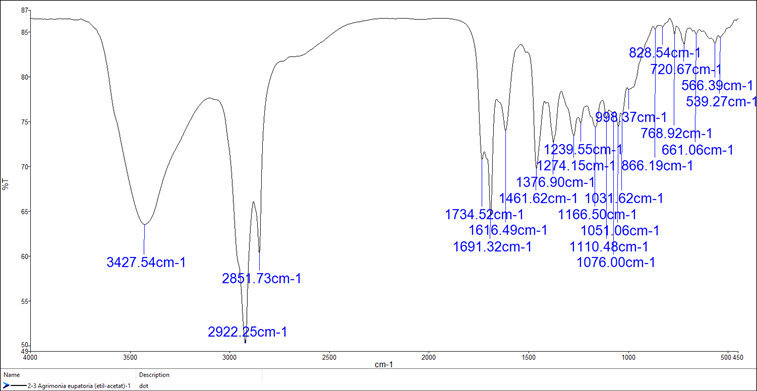
**Fig. S3.** FT-IR spectrum of *A. eupatoria* ethyl acetate extract.


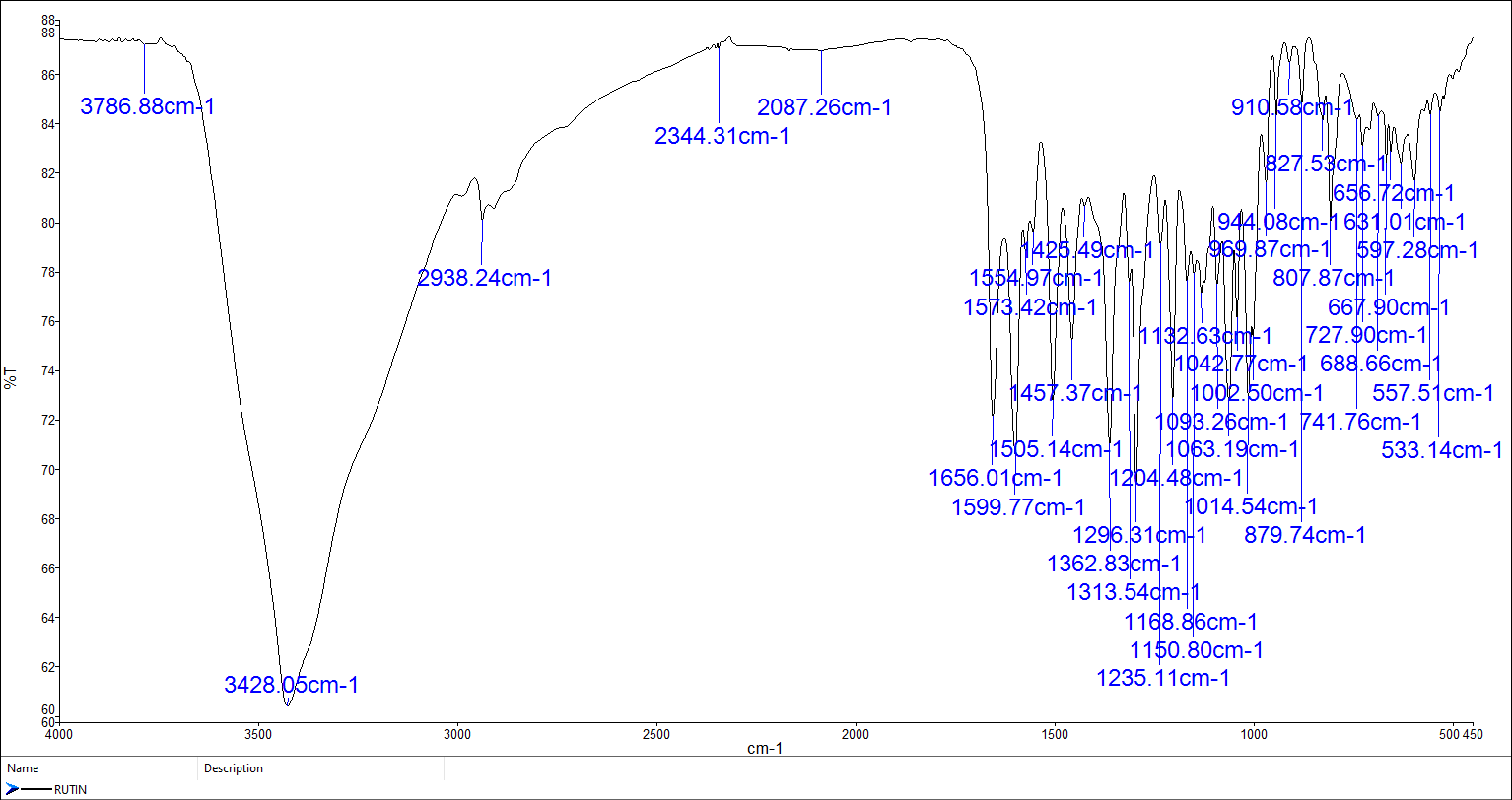

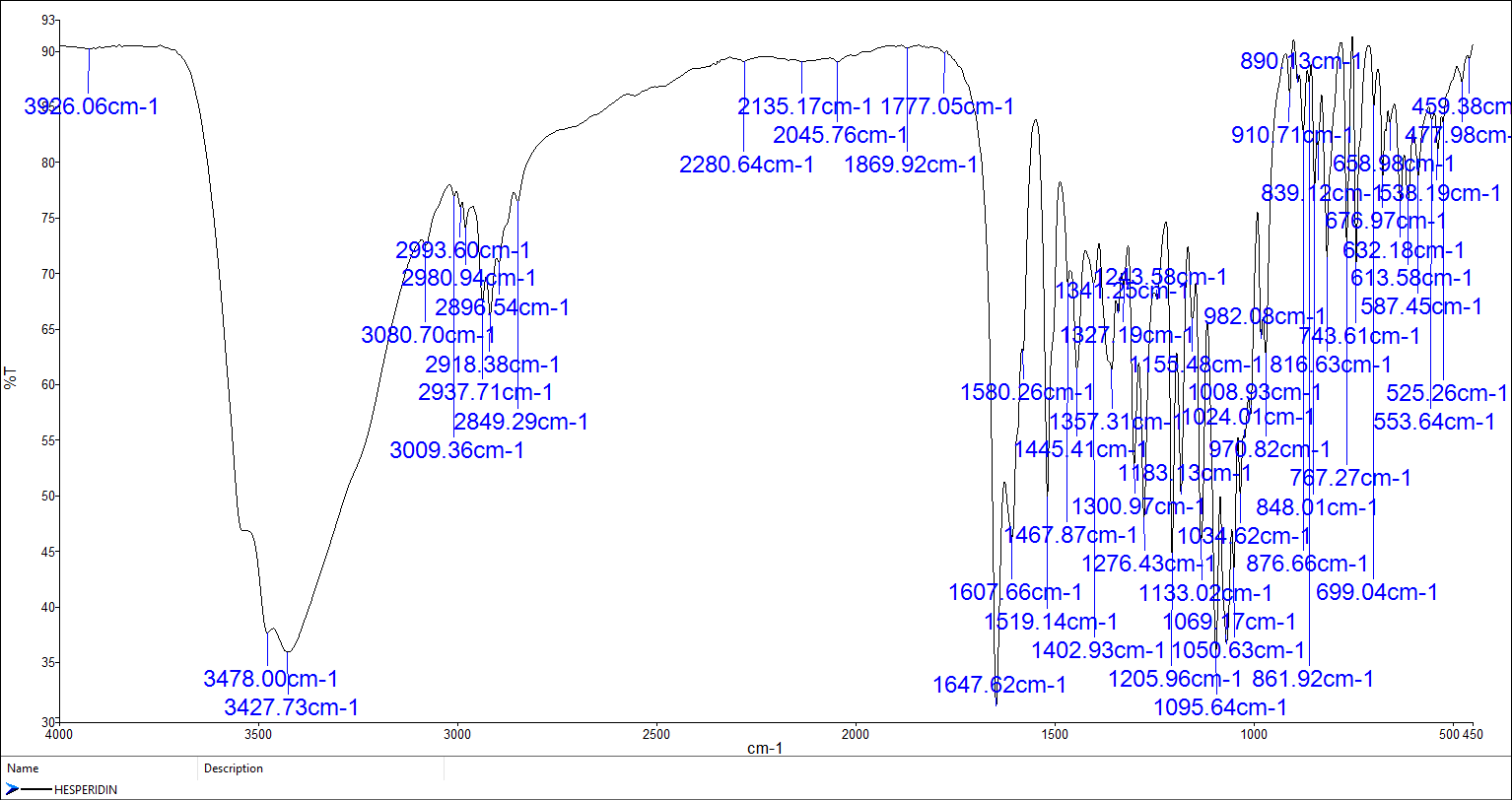

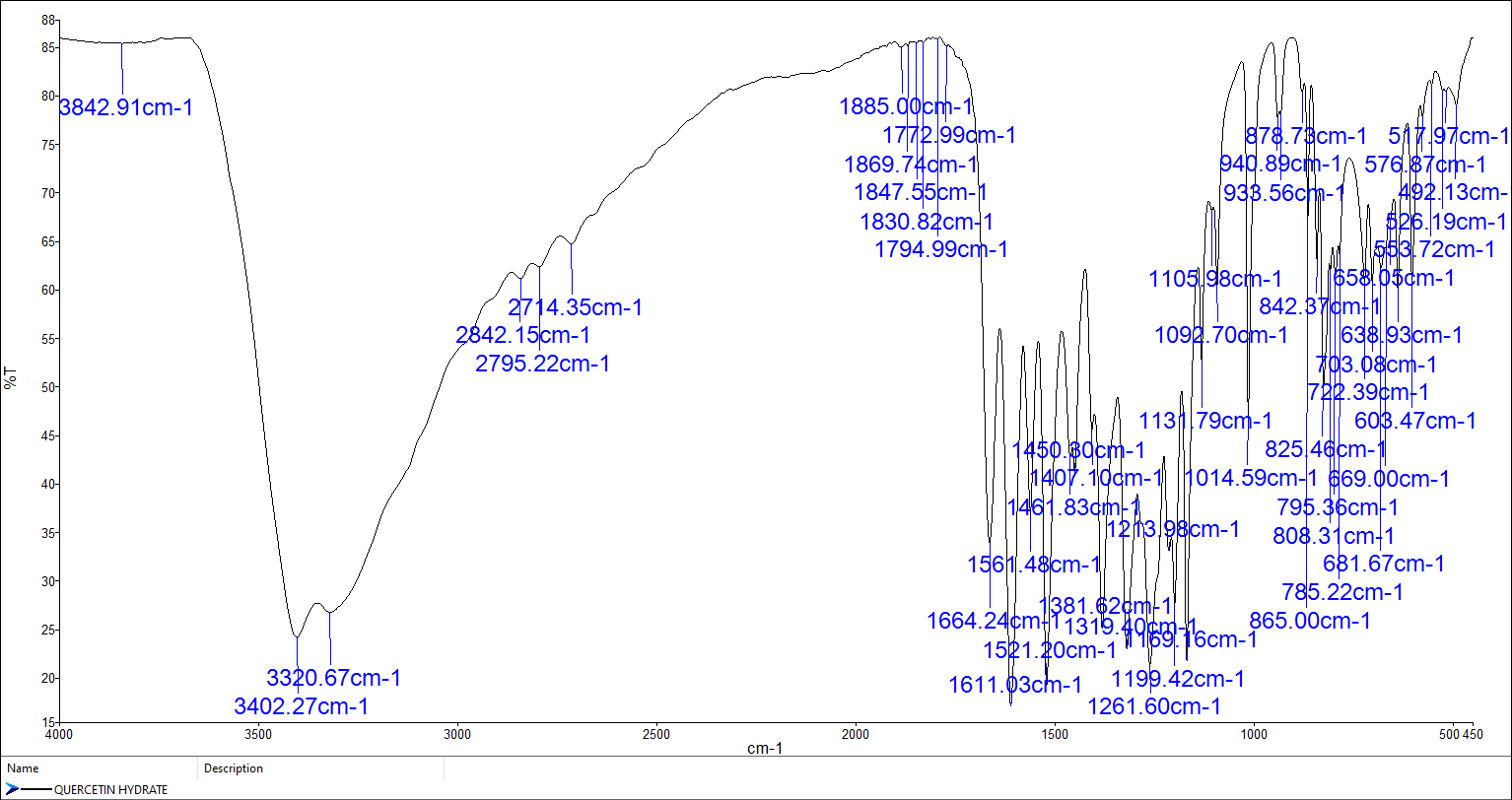


**A**

**B**

**C**

**Fig. S4.** FT-IR spectra of flavonoid standards: A – hesperidin, B – rutin, C – quercetin hydrate.


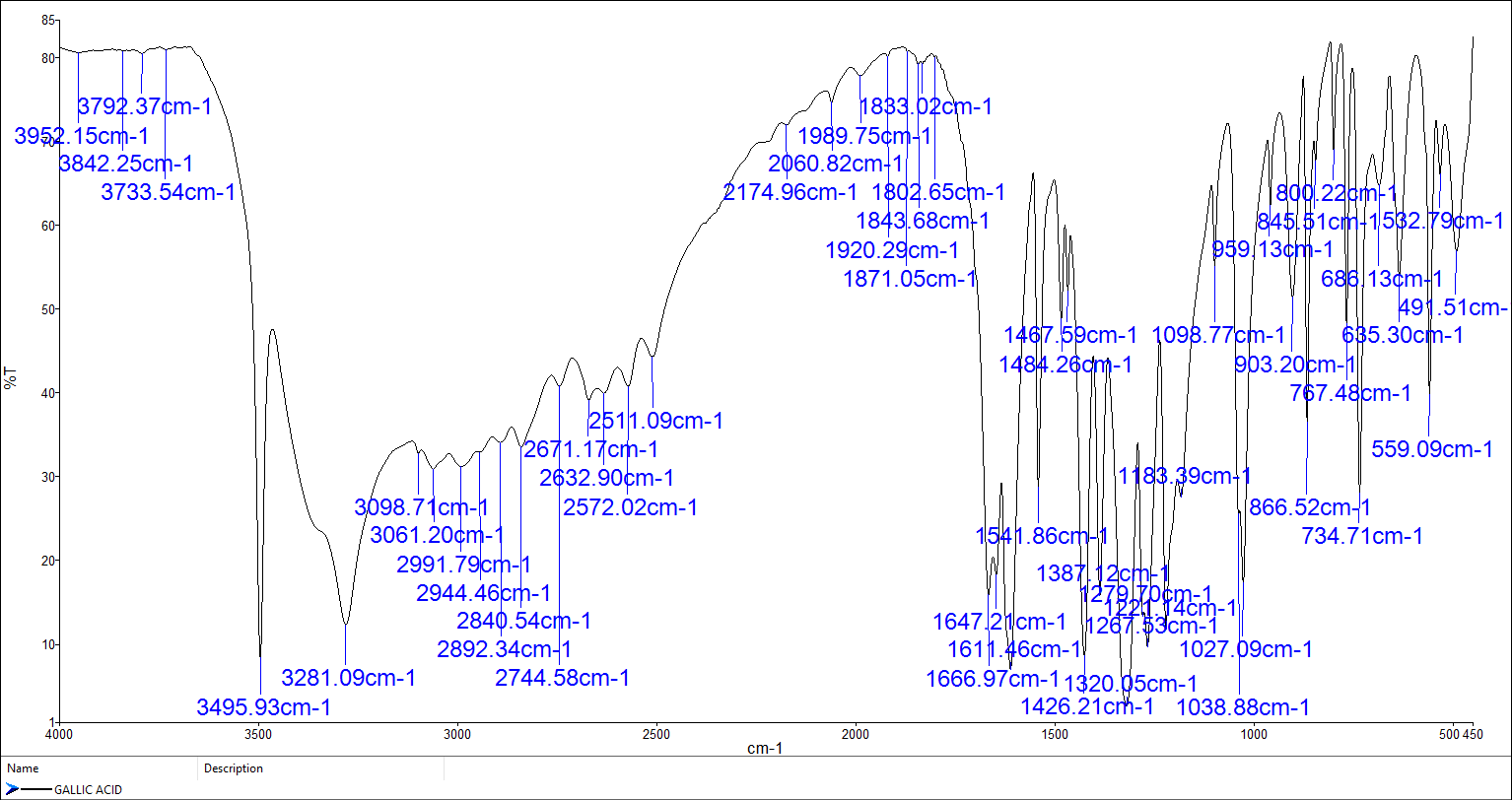

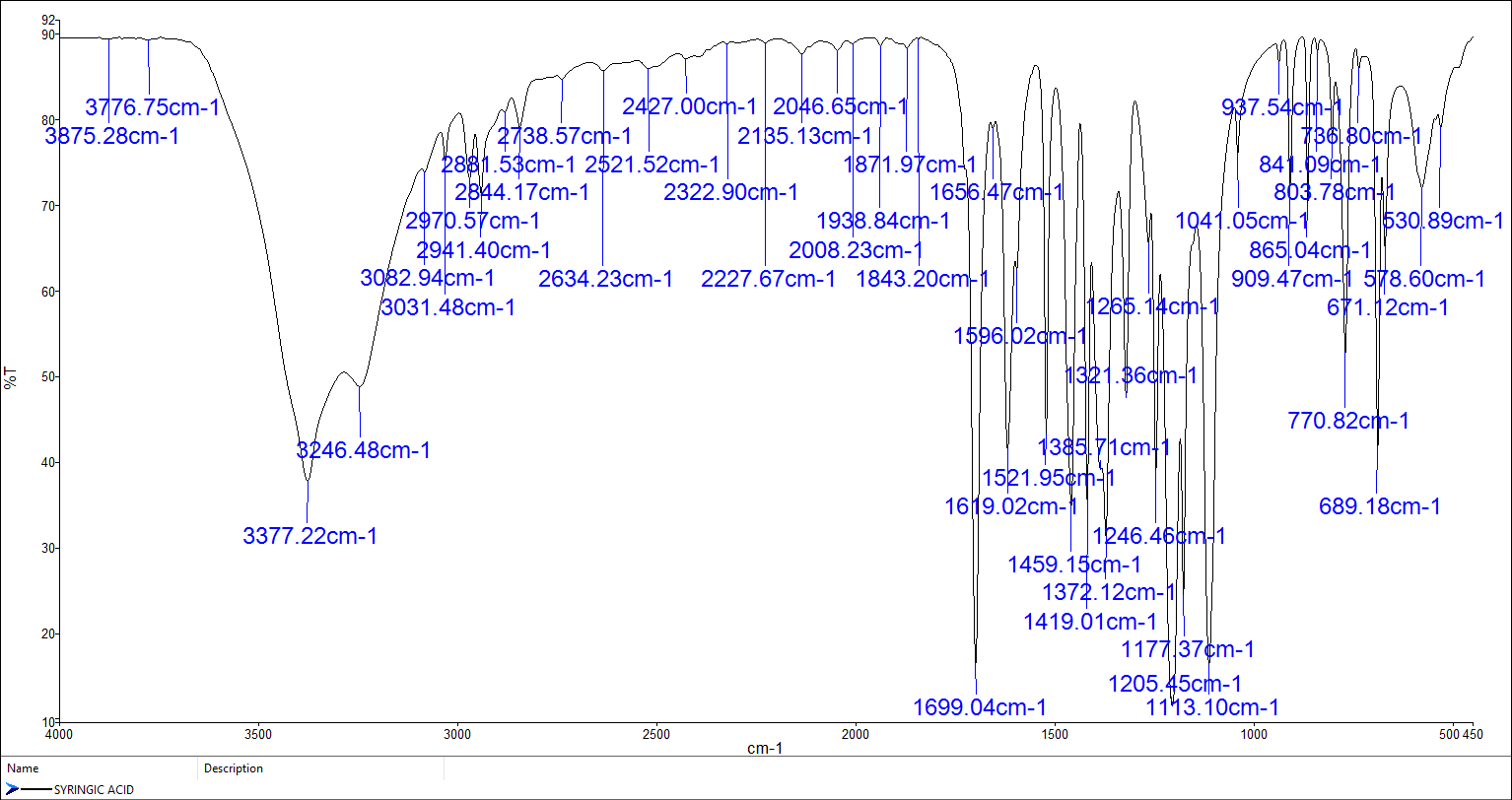

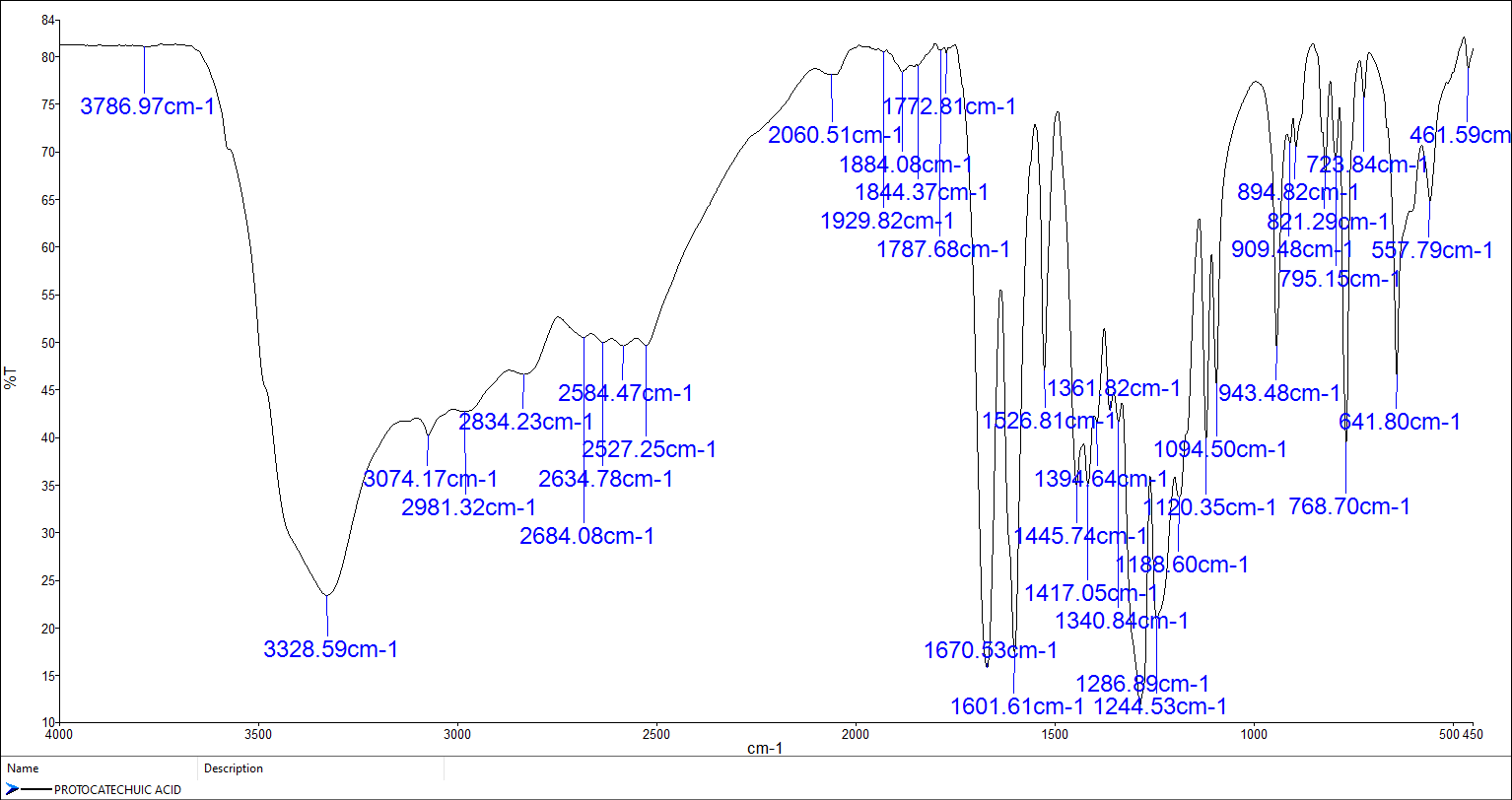


**A**

**B**

**C**

**Fig. S5**. FT-IR spectra of hydroxybenzoic acid standards: A – gallic acid, B – syringic acid, C – protocatechuic acid.


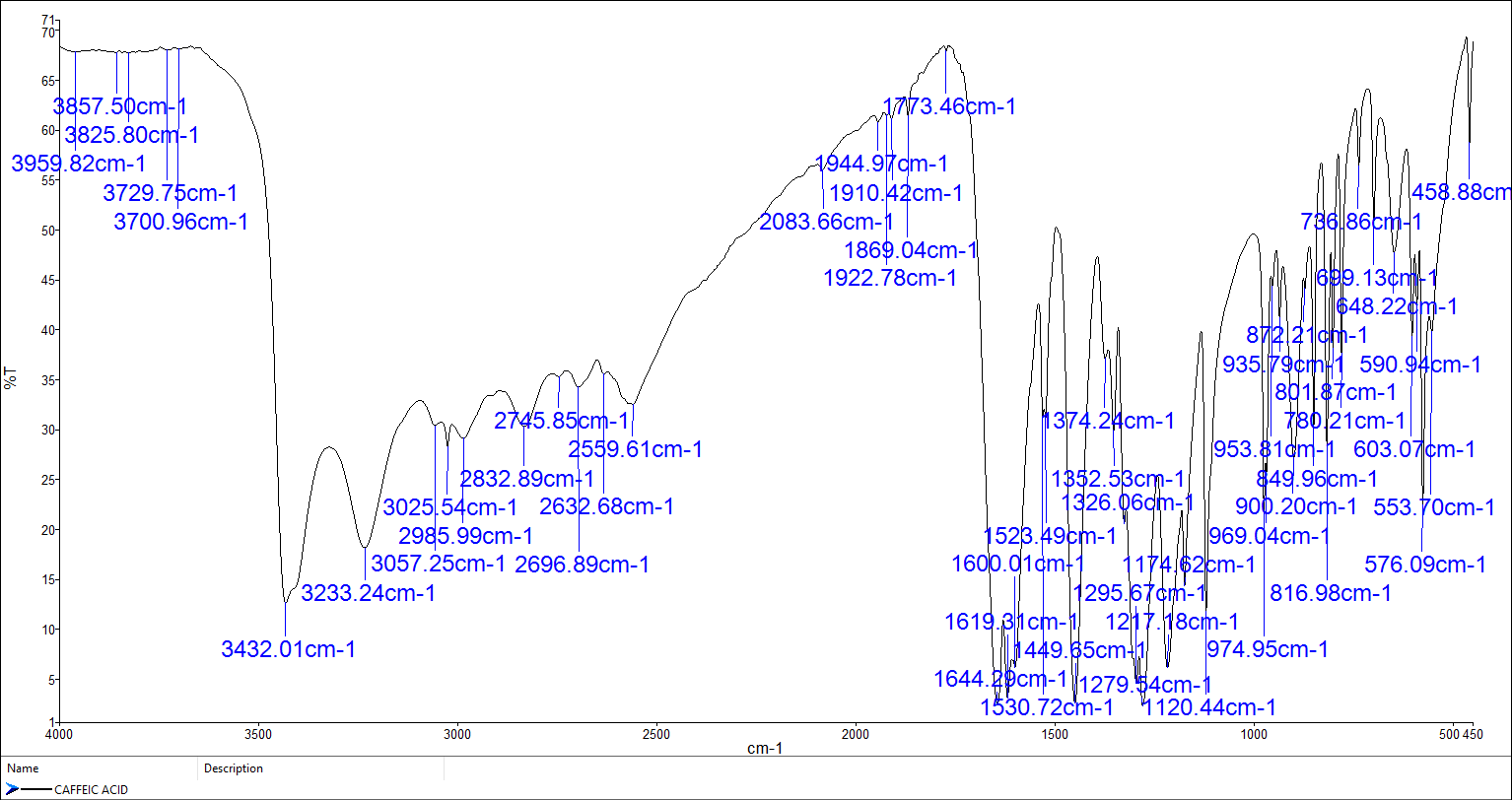

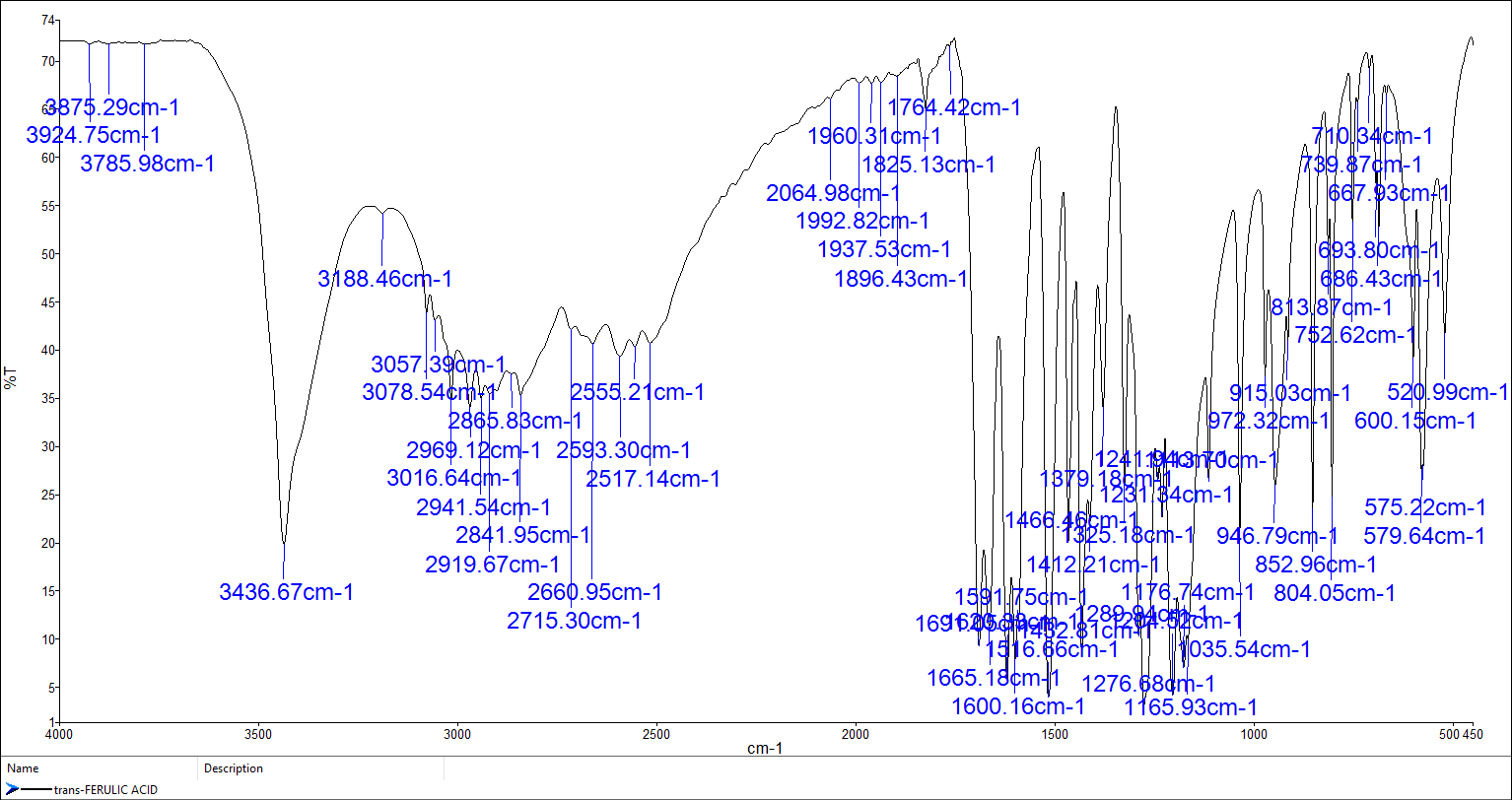


**A**

**B**

**Fig. S6.** FT-IR spectra of hydroxycinnamic acid standards: A – caffeic acid, B – *trans*-ferulic acid.


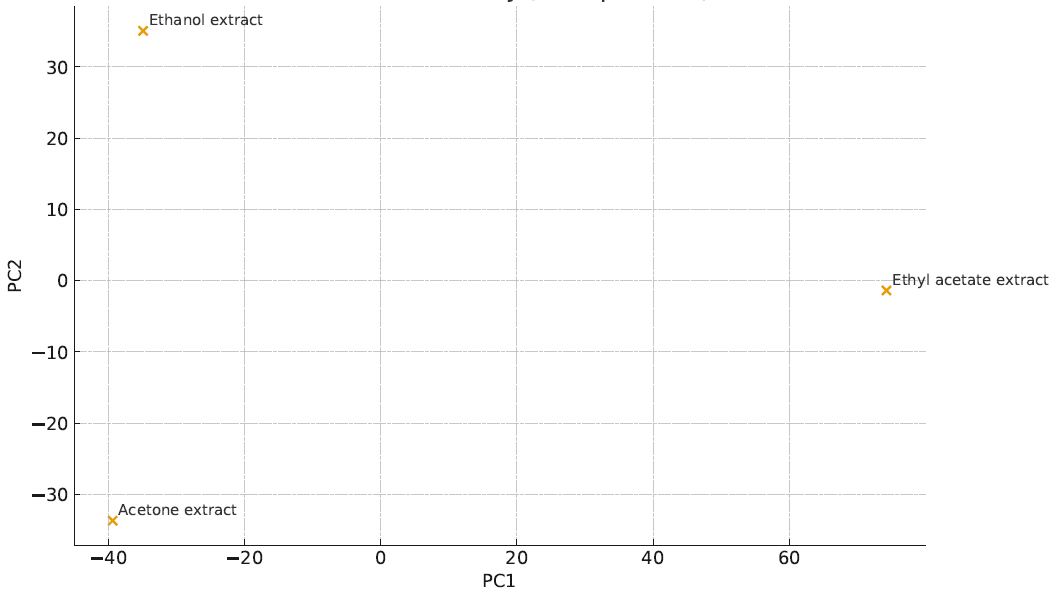


**Fig. S7.** Principal Component Analysis (PCA) score plot of FT-IR spectra. PCA score plot of vector-normalized and autoscaled FT-IR spectra of acetone, ethyl acetate, and ethanol extracts. PC1 (77%) and PC2 (22%) capture all spectral variance, enabling clear separation of extracts based on solvent-dependent chemical composition.


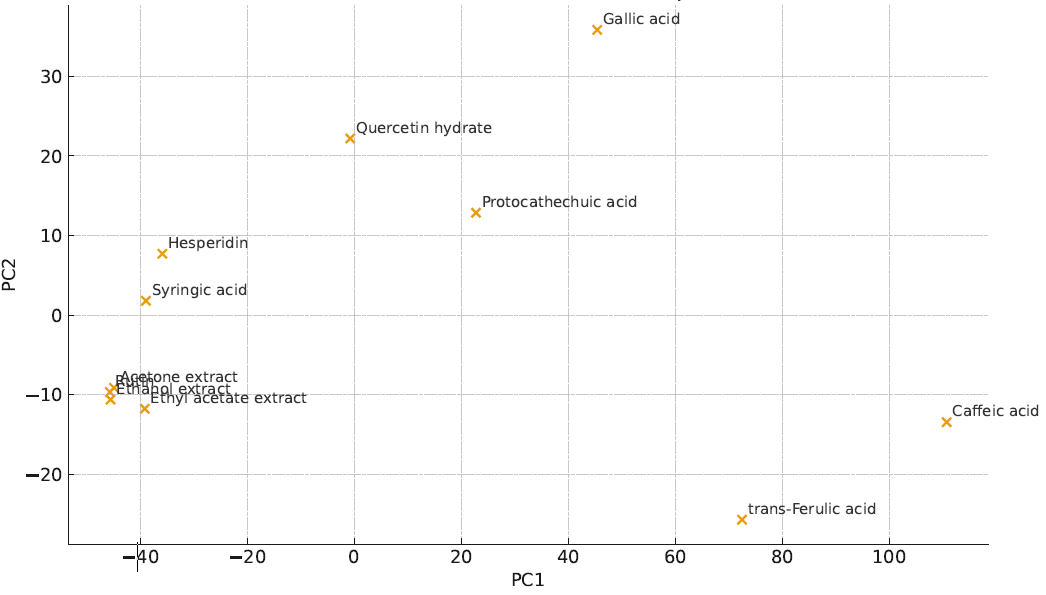


**Fig. S8.** Principal Component Analysis (PCA) score plot of FT-IR spectra. PCA score plot of vector-normalized and autoscaled FT-IR spectra of extracts and standards. PC1 (45.4%) and PC2 (16%) capture all spectral variance, enabling clear separation of extracts based on solvent-dependent chemical composition.


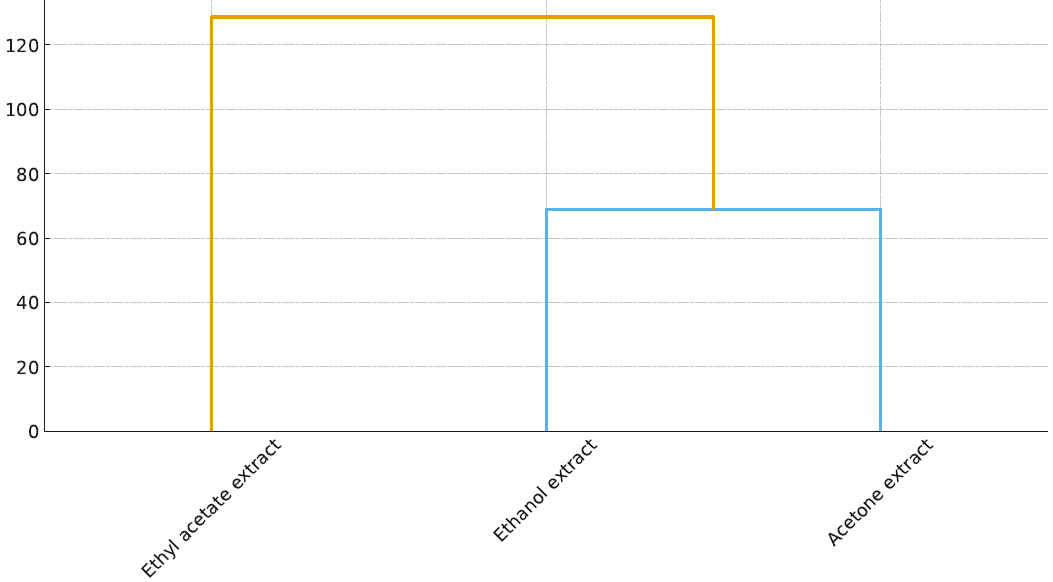


**Fig. S9.** Hierarchical Cluster Analysis (HCA) dendrogram of FT-IR spectra. Dendrogram generated using Ward's linkage and squared Euclidean distance, illustrating clustering patterns among the three extracts. Acetone and ethyl acetate extracts cluster together, while the ethanol extract forms a separate branch, reflecting differences in their FT-IR spectral profiles.


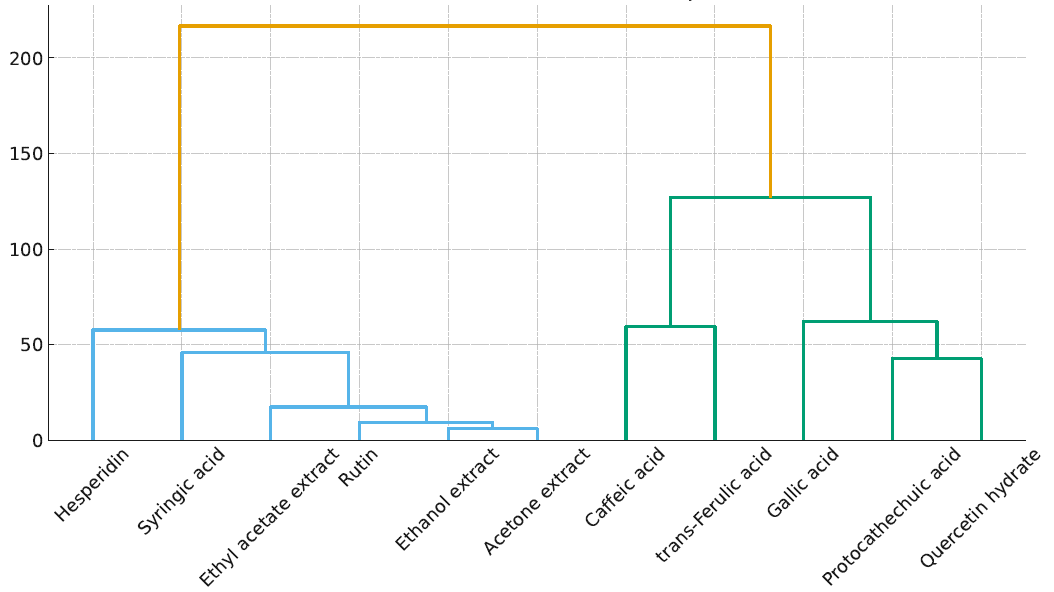


**Fig. S10.** Hierarchical Cluster Analysis (HCA) dendrogram of FT-IR spectra. Dendrogram generated using Ward's linkage and squared Euclidean distance, illustrating clustering patterns among the three extracts and eight standards.


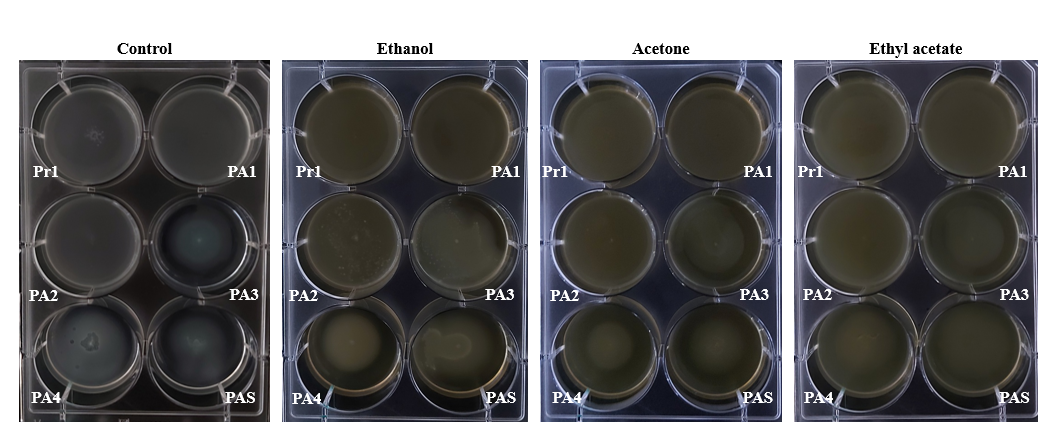


**Fig. S11.** Effect of *A. eupatoria* extracts on swimming motility. (Pr1 *- Proteus* spp.; PA1, PA2, PA3, PA4 - *P. aeruginosa* isolates, PAS - *P. aeruginosa* ATCC 10145).


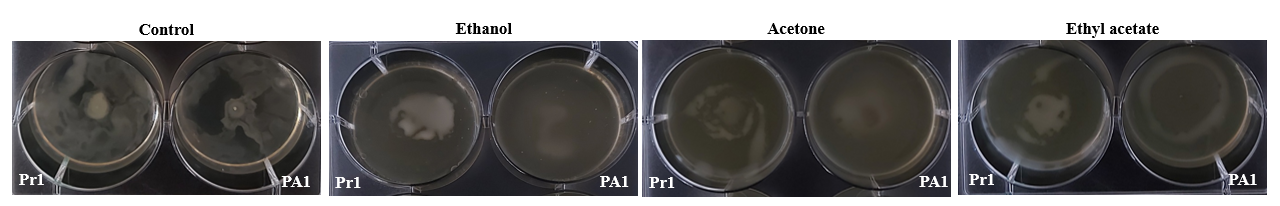


**Fig. S12.** Effect of *A. eupatoria* extracts on swarming motility. (Pr1 *- Proteus* spp.; PA1 - *P. aeruginosa* isolate).
